# Thermodynamically Constrained Averaging Theory: Principles, Model Hierarchies, and Deviation Kinetic Energy Extensions

**DOI:** 10.3390/e20040253

**Published:** 2018-04-05

**Authors:** Cass T. Miller, William G. Gray, Christopher E. Kees

**Affiliations:** 1 Department of Environmental Sciences and Engineering, University of North Carolina, Chapel Hill, NC 27599-7431, USA; 2 US Army Engineer Research and Development Center, Vicksburg, MS 39180-6199, USA

**Keywords:** entropy production, multiscale models, multiphase systems, averaging theory, porous media, turbulent flows

## Abstract

The thermodynamically constrained averaging theory (TCAT) is a comprehensive theory used to formulate hierarchies of multiphase, multiscale models that are closed based upon the second law of thermodynamics. The rate of entropy production is posed in terms of the product of fluxes and forces of dissipative processes. The attractive features of TCAT include consistency across disparate length scales; thermodynamic consistency across scales; the inclusion of interfaces and common curves as well as phases; the development of kinematic equations to provide closure relations for geometric extent measures; and a structured approach to model building. The elements of the TCAT approach are shown; the ways in which each of these attractive features emerge from the TCAT approach are illustrated; and a review of the hierarchies of models that have been formulated is provided. Because the TCAT approach is mathematically involved, we illustrate how this approach can be applied by leveraging existing components of the theory that can be applied to a wide range of applications. This can result in a substantial reduction in formulation effort compared to a complete derivation while yielding identical results. Lastly, we note the previous neglect of the deviation kinetic energy, which is not important in slow porous media flows, formulate the required equations to extend the theory, and comment on applications for which the new components would be especially useful. This work should serve to make TCAT more accessible for applications, thereby enabling higher fidelity models for applications such as turbulent multiphase flows.

## 1. Introduction

Multiphase systems often must be resolved at a length scale that averages over all of the phases present rather than resolving the dynamics of the morphology of the phase distribution. Multiple approaches exist for deriving macroscale models based upon microscale model precursors. Previous work has reviewed three important classes of approaches: the method of volume averaging, averaging conservation equations with rational thermodynamics, and the thermodynamically constrained averaging theory (TCAT) [1]. Other approaches exist as well, such as the Müller and Liu approach developed and applied for multiphase systems [2], which is based on rational thermodynamics. Because we desire models that include phase, interface, common curve, and common point entities and are based on microscale classical irreversible thermodynamics that is averaged to the macroscale, our focus herein is on TCAT.

Modeling of multiphase porous medium systems poses special challenges because of the range of length scales of concern and the need to formulate and close models that are consistent across length scales, while capturing the operative physics with appropriate fidelity. TCAT provides a means to link disparate length scales, produce hierarchies of models of varying complexity, and ensure consistency with the second law of thermodynamics [3,4,5]. The production of entropy due to dissipative processes is used to specify permissibility conditions for constitutive relations needed to produce closed, solvable models. The typical formulation of conservation and balance equations for phases is supplemented in TCAT by inclusion of the corresponding equations for interfaces and common curves. This enables the natural formulation of interfacial transport phenomena and interface behavior. Furthermore, the thermodynamics of phases, interfaces, and common curves, referred to collectively as entities, are posed at the small scale and then averaged to ensure that the expressions obtained are both mathematically rigorous and physically meaningful.

TCAT makes use of a variety of mathematical tools, including general curvilinear coordinate systems, generalized functions, variational methods, change of scale theorems, and differential geometry (e.g., [4,5,6,7,8]). Substantial manipulations are required to arrive at the desired expressions for the entropy density production rate, which we will refer to as entropy inequalities (EIs). The EIs for a given hierarchy of models can span multiple pages of text, which can appear to be impenetrable for the non-specialist.

While the details of the TCAT approach are involved, the guiding principles can be clearly and concisely elucidated to enable the rapid development of high-level understanding. With such an understanding, the substantial quantity of available TCAT results can be leveraged, enabling the formulation of a wide range of closed models with relatively modest manipulations compared to an *ab initio* model-building approach.

To be sure, many additional model formulation, closure, evaluation, and validation issues must be considered to mature fully the TCAT approach. One of the unresolved theoretical issues worth considering is the deviation kinetic energy. The focus to date on TCAT models involving porous medium systems with relatively slow flow has resulted in the treatment of the deviation kinetic energy as a secondary quantity, which is typically removed using a simple thermodynamic system approximation (e.g., [5,9,10,11,12,13]). To extend TCAT models to other systems involving potentially turbulent flows, the deviation kinetic energy could be treated as a leading-order quantity. To do so would require the derivation of conservation equations that have not yet been reported in the literature for lower dimensional entities such as interfaces and curves.

The overall goal of this work is to advance the general understanding and scope of problems accessible using the TCAT approach. The specific objectives of this work are: (1) to present the elements of the TCAT approach to enable a clear conceptual understanding; (2) to summarize the model components that have been derived and can be reused; (3) to illustrate how entropy density production rate equations lead to permissibility conditions for closure relations; and (4) to extend the classes of problems that can be modeled with high fidelity by deriving the necessary deviation kinetic energy equations useful for modeling turbulent transport phenomena in incompressible or compressible systems.

## 2. Elements of the Thermodynamically Constrained Averaging Theory

The purpose of this section is to present some fundamental elements of the TCAT approach to facilitate conceptual and qualitative understanding of the method without delving into the mathematical details. TCAT is useful for deriving multiscale mathematical models of multiphase systems that can contain, in principle, any combination of liquid and solid phases without restriction on the thermo-mechanical properties of these phases. The method assumes that Newtonian continuum physics apply at the smallest scale considered, which we will refer to as the microscale. The desired result is to derive models consistent with known microscale principles at a larger scale, the macroscale, where a “point” is defined as the state of an averaging region known as a representative elementary volume (REV) [14,15,16]. An REV is large enough to contain a representative sampling of all entities in the system. Entities refer to phases, which exist in ${I\;R}^{3}$; interfaces, which form at the boundary between two phases and exist in ${I\;R}^{2}$; common curves, which form at the shared boundary of three phases and exist in ${I\;R}^{1}$; and common points, which form at the shared boundary of four phase and exist in ${I\;R}^{0}$. The scale of the system of concern is the megascale, and TCAT can be used to formulate megascale models as well, where spatial variation within the averaging region is not resolved. TCAT can also produce mixed-scale models in which a three-dimensional system is megascale in one or two dimensions and a smaller scale, say the macroscale, in the remaining dimensions. The key concepts here are thus the nomenclature for the scales involved in a TCAT model, and the objective to start from the microscale to derive a larger scale model that is consistent with the microscale. This consistency implies that if information exists at the microscale, then all macroscale or megascale quantities appearing in the resultant model can be computed precisely and unambiguously.

Two classes of elements of TCAT are discussed: mathematical foundational elements, and model building components. The mathematical foundational elements represent some key concepts and methods used in TCAT to derive the model building components. The mathematical foundational elements are not essential if only a general understanding of TCAT is desired. If one wishes to understand TCAT at a deeper more fundamental level, all the way down to being able to reproduce the available models in complete detail, then the mathematical foundational elements will be of use. The mathematical foundational elements also support a description of how TCAT differs from other common approaches used to formulate continuum models for porous medium systems.

Selected mathematical foundational elements of TCAT are summarized in Table 1. If one considers a multiphase porous medium system at the microscale, the description of entities is challenging because the domain of any single entity can be arbitrarily complex and difficult to describe using a fixed Cartesian coordinate system. General curvilinear coordinate systems [6] are used at the microscale for TCAT to distinguish the outward normal from phases, interfaces, and common curves, and the orientation of a common curve. This set of microscale vectors provides the basis for describing the complex morphology of entities and provides support for differential geometry needed to formulate descriptions of geometric properties such as curvatures and orientations [5]. Curvatures and orientation tensors have analogs at the macroscale, which are needed to support higher fidelity models and geometric-based descriptions of quantities such as the state equation for capillary pressure and preferential flow directions.

**Figure 1:**
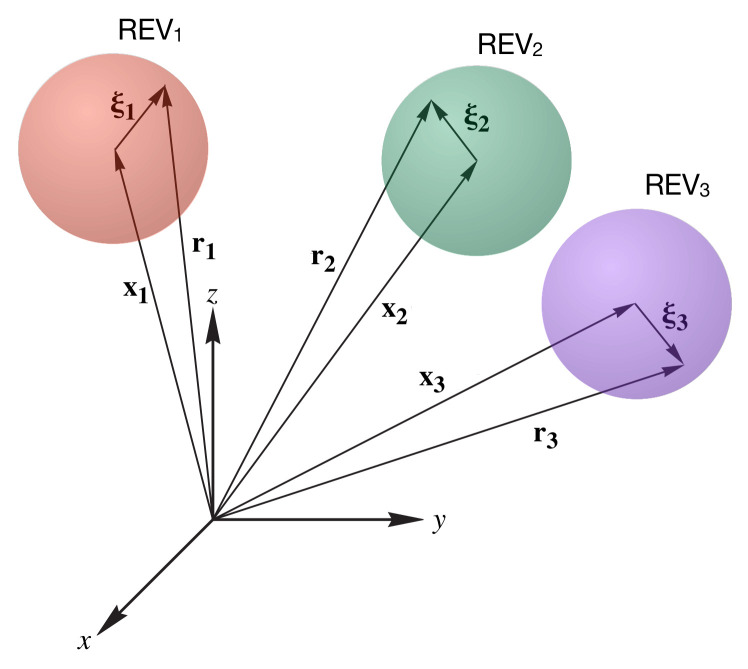
Dual coordinate system used with a fixed x coordinate system and a ξ coordinate system associated with the centroid of an averaging volume.

Some aspects of coordinate systems and averaging operators warrant consideration. A three-dimensional Cartesian coordinate system is typically applicable at the macroscale, as the microscale details of the entity morphology and topology described with curvilinear coordinates at the microscale are averaged. However, the microscale and macroscale coordinates must be described jointly. TCAT uses dual coordinate systems to accomplish this union, as depicted in Figure 1. To fix ideas on the coordinate systems involved, let us consider a microscale variable $f_{\alpha }$ and a macroscale variable $f^{\alpha }$, where α in an entity index with a subscript denoting a microscale quantity, and a superscript denoting a macroscale quantity. In TCAT, the macroscale variable is computed as some precisely-defined average involving microscale quantities. Given the variety of ways in which averages are computed and the need to be precise, some notational complexity results; this cannot be avoided. The way in which various averages are denoted are through adornments to the superscripted variables (e.g., none for an intrinsic average, a single overbar for a density-weighted average, and a double overbar for an explicitly defined average of some other kind). Macroscale averages are defined over some averaging region $\Omega_{}$, which is taken as an REV. We will refer to the spatial location of a point using the position vector r. It is usual in the averaging literature to introduce a decomposition of a microscale quantity into a mean and a fluctuation of the form

(1) $$f_{\alpha }\left(r\right)=f^{\alpha }\left(r\right)+{\tilde{f}}_{\alpha }\left(r\right)\;\mathrm{for}\;r\in \Omega_{\alpha }\;,$$

where an intrinsic average at the macroscale is assumed, and ${\tilde{f}}_{\alpha }$ is the deviation between the microscale value and the average. This decomposition can only be specified when r is a point in the α entity because microscale values of $f_{\alpha }$ do not exist except in that entity. With this decomposition, all quantities are specified uniquely at a coordinate location. Formulating the deviations in this way is standard, for example, in the method of volume averaging (e.g., [20]) or turbulence analyses when averaging is over time. With TCAT, the decomposition is formulated in terms of both a macroscale coordinate x, and a microscale coordinate ξ. The macroscale coordinate locates the centroid of the REV and ξ is a position vector relative to an origin located at x, as in Figure 1. The formulation for the decomposition analogous to Equation (1) is thus

(2) $$f_{\alpha }\left(x+\xi \right)=f^{\alpha }\left(x\right)+{\tilde{f}}_{\alpha }\left(x,\xi \right)\;\mathrm{for}\;x+\xi \in \Omega_{\alpha }\;.$$

This difference in decomposition is subtle, yet important. The deviation quantity, ${\tilde{f}}_{\alpha }$, is a function of both x and ξ and not just of their sum. By making use of a dual coordinate system, the TCAT decomposition ensures that the average of the deviations must vanish over an REV. In turbulence, this is sometimes referred to as an optimal filter, and it cannot be assured using the approach of Equation (1). This concept extends to different sorts of averages as well.

Typically in modeling multiphase systems, models are based on phases alone and jump conditions are used to account for exchange of conserved or balanced quantities between phases. TCAT diverges from this common approach by formulating dynamic conservation and balance equations for all entities (phases, interfaces, common curves, and common points) such that all entities have properties themselves. This approach has been shown to enable higher fidelity models [21]. This approach also produces models with more equations that must be closed and solved.

Another complication resulting from the inclusion of the lower-dimensional entities is that an REV, $\Omega_{}$, contains a set of entities of varying dimensions that require theorems that allow the exchange of the order of differential and integral operations. Unlike the simple case of averaging over a time interval or of spatial averaging of microscale system consisting of a single phase, spatial differentiation and integration cannot simply be exchanged with multiphase systems due to boundaries between entities within the averaging volume. Resolution of this issue requires the formulation of theorems that provide a transformation between scales for phases, interfaces, common curves, and common points. To derive the needed theorems, generalized functions can be used to enable integration over the domain even for lower dimensional entities; these functions vanish from the final result. Thus, they are a mathematical tool that provides an alternative, and perhaps simpler, way of deriving formal change of scale theorems relative to other approaches. A large number of such theorems have been derived and are available in the literature [5,6].

Thermodynamics enables the description of equilibrium states of matter. Certain extremum principles are known to exist at equilibrium, such as a state of minimum energy and maximum entropy. While classical irreversible thermodynamics can be developed for phases (fluids and solids), interfaces, and common curves, conditions that must hold for systems that contain some combination of these variable dimension entities are not obvious, even in light of the guiding extremum principles. TCAT formulates expressions for entropy production rate densities of a form that involves the sum of the product of fluxes and forces, such that all fluxes and forces vanish at equilibrium. To identify these sets of fluxes and forces and guide the formulation of the desired entropy inequality (EI), it is useful to know all conditions that must hold at equilibrium. To derive these conditions, TCAT relies upon variational methods.

In general, one can write

(3) $$F=\int_{\Omega_{}}f_{n}\left(u\right)\;dr\;$$

where *F* is the quantity to be minimized, $f_{n}$ is a functional, and u is a vector of unknown functions. Variational methods can be used to solve this minimization problem, thus satisfying extremum conditions for *F* and identifying the form of the functional $f_{n}$. Thus, for a given prescription of the entities in a system, one is able to deduce the conditions that must hold at equilibrium. These equilibrium results provide insights into the optimal use of an EI for the system in deriving closure relations.

Geometry plays a crucial role in the description of physical phenomena [22]. This is certainly the case for multiphase porous medium systems even in the limit of strictly Newtonian continuum physics, where the added complexities of quantum and relativistic effects can be ignored. The geometric aspects of such systems involve the description of curvatures of interface and common curve entities and the orientation of these entities in ${I\;R}^{3}$. Differential geometry enables the derivation of invariants that reduce the description of the geometric aspects of a system to their simplest possible form. Recent work has shown that capillary pressure can be described in terms of invariants related to measures of volume, area, mean curvature, and the Gaussian curvature [23]. Such a state equation has the benefit of applying under both equilibrium and dynamic conditions and being supported by established theorems from topology. TCAT relies upon differential geometry to formulate the invariants needed to provide a robust geometric description of the state of a system.

**Figure 2:**
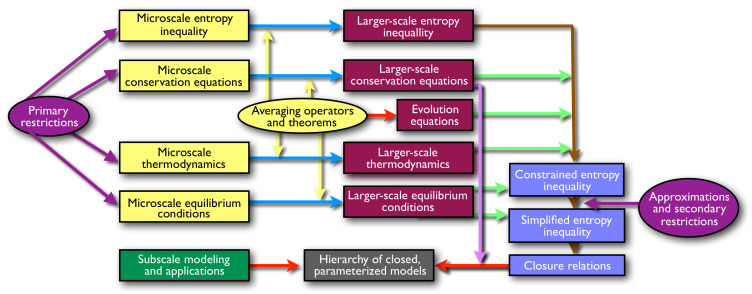
Schematic representation of the TCAT model formulation approach.

The mathematical foundations underpin a set of components shown in Figure 2 that are combined to formulate closed, solvable models of transport phenomena in a wide variety of systems. Figure 2 also shows the general progression of model development, which is denoted by the arrows. Note that arrows terminate at the desired endpoint shown on the bottom middle of the figure, a closed solvable model at a larger scale (e.g., macroscale). TCAT integrates many components in producing a model. Conservation and balance equations are written for each entity, or for the chemical species in the entity, at the microscale. A set of thermodynamic equations are also written for each entity, and a set of conditions that must hold at equilibrium are deduced using variational methods. An averaging operator is applied to the conservation and thermodynamic equations to derive larger-scale equations. Averaging theorems are used to exchange the order of differentiation and integration when averaging microscale equations over an REV, yielding a set of accessible quantities with minimal cardinality. The macroscale entropy balance equation is arranged to solve for the non-negative entropy density production rate by summing over all entities. The problem that arises is that this entropy production expression is not explicitly connected to the physical processes that produce entropy. To make this connection, TCAT augments the entropy production equation with the sum of Lagrange multipliers multiplying each of the macroscale conservation equations for mass, momentum, and energy for all entity types as well as the expression for thermodynamics and body force potentials. Because these equations are all arranged to be zero, they do not alter the magnitude of the rate of entropy production. Augmentation with the conservation equations enables the derivation of an expression that relates the sum of products of fluxes and forces to the non-negative entropy production rate. The form closest to the flux–force form that can be derived is referred to as the constrained entropy inequality (CEI). Approximations are needed to yield a strict flux–force form of the EI, which is referred to as a simplified entropy inequality (SEI). The SEI is then used to develop approximations for each of the fluxes in terms of the forces. These approximations must be consistent with the SEI. The conservation equations and closure approximations do not form a closed set of equations under general conditions. This is because in multiphase systems entity extent measures such as volume fractions, specific interfacial areas, and specific common curve lengths are included. Evolution equations based purely on the averaging theorems and simplifying approximations are used to produce the needed geometric equation balances required for a closed model [5,24].

Because of the scale consistency of TCAT, all macroscale quantities can be computed from the microscale dynamics of the system. Increasingly, pore-scale experimental methods and modeling are being used to provide detailed values of these variables that can be averaged to obtain their macroscale counterparts. With this macroscale information, the approximations used to posit a macroscale TCAT model and the solutions of the macroscale equations can be assessed, general state equations can be reduced to specific forms, and closure parameters, and their functional dependence, can be examined in detail. Symbiotically, the detailed microscale information and the derived macroscale TCAT models can be employed to obtain robust, closed, and validated governing equations.

## 3. Existing Hierarchies of Models

Many small-scale elements of TCAT are available and can be used as a basis for the derivation of consistent larger scale models. For example, all microscale conservation and balance equations for all entities are available, all change of scale theorems needed have been derived, complete microscale thermodynamic equations based upon classical irreversible thermodynamics and potential equations exist. The averaging of these equations directly yields their macroscale counterparts. Additionally, a spectrum of macroscale evolution equations for geometric quantities can be obtained from the averaging theorems. If one wishes to derive models for various physical systems, these macroscale components can be used directly rather than having to rederive them. This results in a substantial savings of effort. An even more efficient way to develop TCAT models is to leverage the existing hierarchies of models.

As indicated in Figure 2, the usual approach for TCAT model formulation is to make a set of primary restrictions that specify the general entities and compositional aspects of a model. With conservation equations for these entities known, a thermodynamic theory, and the requirement that an REV exist such that the averages defined are mathematically well behaved allows one to proceed. The primary restrictions are a balance between a desire to produce a completely general model framework and the desire to keep the analysis from being onerously complex. On one extreme, the primary restrictions could be so extensive as to admit only one very specific model (e.g., [25]). On the other extreme, the primary restrictions could be so general as to produce a model for a very wide range of systems such that most applications would be some subset of the derived formulation. Neither of these two extremes is optimal in our view. Formulating a single specific model using TCAT is inefficient because of the significant manipulations involved. If the model is found to be inadequate, the entire process would need to be repeated. On the other hand, the complexity of the formulation process grows with the complexity of the system making the derivation of the necessary SEI increasingly burdensome as the number of phases, compositional characteristics, and complexity of the transport phenomena increase. An all-inclusive model would require a lengthy derivation, both in time and space needed to write the equations, which would be unnecessary for simpler systems. As the number of entities increases, the need for geometric evolution equations increases; these equations are challenging expressions to develop. Thus, if a system of interest contains few entities, the derivation of the TCAT model is easier to accomplish.

TCAT work to date has pursued a middle ground between the extremes of complexity and simplicity. General hierarchies of specific classes of models have been targeted in the development of EIs. These classes of models can be identified by the thermodynamic theory, the entities considered, the compositional components modeled, the scale of the resultant model, and operative processes. Even with specification of each of these aspects of a model identified, a hierarchy of potential models results that can serve many potential applications. This hierarchy results for several reasons. First, the primary restrictions will lead to a CEI, which is essentially exact. However, approximations are needed to produce an SEI; these approximations are subject to change, and hopefully improvement, as new insights and methods become available. Changing the SEI will result in a change in the permissibility conditions for the closure relations, which will echo through the model closure. Second, a set of secondary restrictions may be applied to a general SEI to simplify its form to a restricted SEI. As an example, if after deriving a general SEI one wishes to consider only isothermal systems with no mass or internal energy exchange between entities, a much simpler SEI will result as some of the terms will go to zero. Alternatively, one might choose to neglect some lower dimensional entities or ascribe certain properties to an entity. For example, a common simplification is that interfaces and common curves are considered to be massless. Such choices reduce the SEI to a more condensed form but do not require a complete reformulation of the model. Third, whatever the form of the SEI, it only provides permissible constraints on closure relations. The closure relations that are proposed from an SEI can be conjugate flux–force or cross-coupled in form; they may also vary in their order of the approximation. Thus, multiple sets of closure relations, and specific model instances, can be derived from a single SEI. Stated concisely, hierarchies of models result because a given CEI can yield a set of SEIs and a given SEI can yield a set of different closure relations; the cardinality of the sets results in a hierarchy of models of varying sophistication. Model sophistication can be matched to a given application, without the need to completely reformulate the model from scratch.

Because substantial TCAT work has been accomplished in recent years, many hierarchies of models are available, which comprise a resource that can be drawn upon for rapid model building. While significant mathematical machinery and manipulations are required to derive the EI expressions that form the bases of the model hierarchies, it is not necessary to understand the details of the mathematical manipulations that led to these expressions in order to use the results productively. Table 2 summarizes hierarchies of models that have been derived. These substantial results form the bases for a large number of specific instance models that can be formulated with minimal effort.

## 4. Deviation Kinetic Energy

### 4.1. Overview

While existing TCAT results can be used and leveraged to derive models for a variety of systems, further theoretical advancements are needed to address new classes of models. The goal of this section is to illustrate how new TCAT components can support the development of new hierarchies of models. This is accomplished by identifying a class of model of interest, assessing the additions needed to existing TCAT components, deriving certain aspects that are needed to serve as both an example of the details of the model component formulation process and to serve as an archival source of the derived results for future use.

Consider the case of sediment transport in shallow waters that may be turbulent. This system consists of a solid phase and two fluid phases, air and water. At first glance, this seems to be similar to application types considered previously using TCAT. However, some aspects of this problem deserve further consideration. First, with TCAT, and other continuum mechanical approaches, it is necessary to reference all velocities to a common frame of reference. For small Reynolds number porous medium applications considered to date, this reference velocity has been the solid-phase velocity, due to its very small magnitude relative to the fluid. This frame of reference is not a reasonable choice for sediment transport where the solid particle velocity is of the same order of magnitude as the fluid velocities. This means material derivative expressions appearing in the formulation must be changed from a reference to the solid phase to a more appropriate frame of reference. Multiple choices exist for such a choice, and the manipulations to change from a solid-phase reference to another reference velocity, while tedious, are straightforward and will not be detailed here. Second, for the applications considered to date a term related to the average of the product of deviation velocities between the macroscale and microscale has been considered of lower order importance and not specifically considered in formulating the EI. Specifically, the term not considered is the kinetic energy of the deviation velocities per unit mass. For turbulent flow, this term that arises in the energy equation cannot be neglected. A way in which this effect can be included is to formulate deviation kinetic energy equations for phases, interfaces, and common curves that are applicable under general conditions such as compressible or incompressible flows and a specified stress tensor for the entities. The formulation of these equations are detailed below and contribute to the archival model building components.

### 4.2. Macroscale Deviation Kinetic Energy for a Phase

The purpose of this section is to formulate macroscale deviation kinetic energy equations for a phase. We will manipulate and average conservation equations for conservation of momentum and mass at the microscale to formulate this equation. No assumptions will be required. Therefore, the resultant expressions will be applicable for both incompressible and compressible conditions. We also will not assume a form of the stress tensor at either the microscale or the macroscale. Closure relations can be substituted into the final forms as needed.

We can write the microscale velocity as

(4) $$v_{\alpha }\left(x+\xi \right)=v_{}^{\bar{\alpha }}\left(x\right)+{\tilde{v}}_{\alpha }\left(x,\xi \right)\;,$$

and the macroscale fluctuation kinetic energy density as

(5) $$\epsilon^{\bar{\bar{\alpha }}}\rho^{\alpha }K_{E}^{\bar{\bar{\alpha }}}\left(x\right)={\langle \rho_{\alpha }K_{E\alpha }\rangle }_{\Omega_{\alpha },\Omega_{}}={\langle \rho_{\alpha }\frac{{\tilde{v}}_{\alpha }\cdot {\tilde{v}}_{\alpha }}{2}\rangle }_{\Omega_{\alpha },\Omega_{}}\;,$$

where $\epsilon^{\bar{\bar{\alpha }}}$ is the volume fraction, $\rho^{\alpha }$ is the mass density, $K_{E\alpha }$ and $K_{E}^{\bar{\bar{\alpha }}}$ are the microscale and macroscale deviation kinetic energies per unit mass, respectively; α is a phase index; and ${\langle \cdot \rangle }_{\Omega_{\alpha },\Omega_{}}$ is a spatial averaging operator. A subscripted index denotes a microscale quantity and a superscripted index denotes a macroscale quantity.

The macroscale deviation kinetic energy described in Equation (5) is a quantity that arises as a result of applying an averaging operator to the kinetic energy term in a conservation of energy equation. Because it is desirable to express averages in terms of macroscale averaged quantities, a decomposition of the sort described in Equation (4) can be used to convert expressions to resolvable independent expressions of mean quantities and an unknown product of deviations from the mean. The deviations from the mean result from the nature of averaging a microscale quantity to the macroscale. A complex velocity field at the microscale is averaged to produce a single mean velocity. The existence of any solid phase not moving with the same velocity as the fluid (e.g., a fixed porous medium, or a relatively large dense particle settling in a water column) will necessarily result in a microscale velocity that differs from an averaged macroscale velocity. For many porous medium systems, the averaged product of these velocity deviations are small, and resultantly the macroscale deviation kinetic energy term is often neglected (e.g., [11]). For cases of complex flow fields with a wide range of velocities over an averaging region, the macroscale deviation kinetic energy is not small. We wish to derive an expression for the deviation kinetic energy that can be applied to such cases.

The averages discussed here are spatial averages, which is the standard approach used to derive TCAT models [4]. Temporal and ensemble averages are often used in the fluid mechanics literature as well [26]. Under conditions of ergodicity, macroscale models are invariant in form with respect to the type of averaging used to derive these models, although the underlying definitions of variables must be expressed in terms of the averaging approach relied upon. While volume averaging has been used routinely for multiphase systems to derive averaged macroscale models, the scale over which averaging is performed must yield well-defined averages for all quantities in the model that are not sensitive to the size of the averaging region, which we will call a representative elementary volume (REV). If the resultant model can be solved at the REV scale, then only closure of the macroscale model is needed. However, for cases in which the upper bound on the size of REV exists due to significant changes in the underlying quantities, a second level of averaging can be applied—resulting in a so-called doubly-averaged model. The second level of averaging can be of different form than the initial averaging. For example, volume averaging can be followed by time averaging. Doubly-averaged systems result in the need for additional closure relations when compared to singly-averaged models. Doubly-averaged formulations are relatively common in the literature (e.g., [27,28]). In this work, we will focus only on volume-averaged quantities over an REV. The resultant expressions will then be of use for direct numerical simulation done at the macroscale, or as a starting point for a multiply-averaged model.

The microscale conservation of mass equation for a phase is

(6) $$M_{\alpha }:=\frac{\partial \rho_{\alpha }}{\partial t}+\nabla \cdot \left(\rho_{\alpha }v_{\alpha }\right)=0\;\mathrm{for}\alpha \in I_{P}\;,$$

where $I_{P}$ is the index set of all phases. The microscale conservation of momentum equation for a phase is

(7) $$P_{\alpha }:=\frac{\partial \left(\rho_{\alpha }v_{\alpha }\right)}{\partial t}+\nabla \cdot \left(\rho_{\alpha }v_{\alpha }v_{\alpha }\right)-\nabla \cdot t_{\alpha }-\rho_{\alpha }g_{\alpha }=0\;\mathrm{for}\alpha \in I_{P}\;,$$

where $t_{\alpha }$ is the stress tensor, and $g_{\alpha }$ is the gravitational acceleration vector.

Equation (7) can be simplified using Equation (6) to the expression

(8) $$P_{\alpha }-v_{\alpha }M_{\alpha }=\rho_{\alpha }\frac{\partial v_{\alpha }}{\partial t}+\rho_{\alpha }v_{\alpha }\cdot \nabla v_{\alpha }-\nabla \cdot t_{\alpha }-\rho_{\alpha }g_{\alpha }=0\;,$$

which can be written using Equation (4) as

(9) $$\rho_{\alpha }\frac{\partial }{\partial t}\left(v_{}^{\bar{\alpha }}+{\tilde{v}}_{\alpha }\right)+\rho_{\alpha }v_{\alpha }\cdot \nabla \left(v_{}^{\bar{\alpha }}+{\tilde{v}}_{\alpha }\right)-\nabla \cdot t_{\alpha }-\rho_{\alpha }g_{\alpha }=0\;.$$

Taking the dot product of Equation (9) with ${\tilde{v}}_{\alpha }$ and expanding terms yields

(10) $$\begin{array}{cc} {\tilde{v}}_{\alpha }\cdot \left(P_{\alpha }-v_{\alpha }M_{\alpha }\right)= & \rho_{\alpha }{\tilde{v}}_{\alpha }\cdot \frac{\partial v_{}^{\bar{\alpha }}}{\partial t}+\rho_{\alpha }{\tilde{v}}_{\alpha }\cdot \frac{\partial {\tilde{v}}_{\alpha }}{\partial t}+\rho_{\alpha }v_{\alpha }\cdot \left(\nabla v_{}^{\bar{\alpha }}\right)\cdot {\tilde{v}}_{\alpha }\\ & +\rho_{\alpha }v_{\alpha }\cdot \left(\nabla {\tilde{v}}_{\alpha }\right)\cdot {\tilde{v}}_{\alpha }-\left(\nabla \cdot t_{\alpha }\right)\cdot {\tilde{v}}_{\alpha }-{\tilde{v}}_{\alpha }\cdot \rho_{\alpha }g_{\alpha }=0\;, \end{array}$$

which can be reexpressed as

(11) $$\begin{array}{c} \rho_{\alpha }{\tilde{v}}_{\alpha }\cdot \frac{\partial v_{}^{\bar{\alpha }}}{\partial t}+\rho_{\alpha }\frac{\partial }{\partial t}\left(\frac{{\tilde{v}}_{\alpha }\cdot {\tilde{v}}_{\alpha }}{2}\right)+\rho_{\alpha }v_{\alpha }\cdot \left(\nabla v_{}^{\bar{\alpha }}\right)\cdot {\tilde{v}}_{\alpha }\\ +\rho_{\alpha }v_{\alpha }\cdot \nabla \left(\frac{{\tilde{v}}_{\alpha }\cdot {\tilde{v}}_{\alpha }}{2}\right)-{\tilde{v}}_{\alpha }\cdot \left(\nabla \cdot t_{\alpha }\right)-{\tilde{v}}_{\alpha }\cdot \rho_{\alpha }g_{\alpha }=0\;. \end{array}$$

With the microscale deviation kinetic energy per unit mass defined as

(12) $$K_{E\alpha }:=\frac{{\tilde{v}}_{\alpha }\cdot {\tilde{v}}_{\alpha }}{2}\;,$$

Equation (11) is alternatively written as

(13) $$\begin{array}{c} \rho_{\alpha }{\tilde{v}}_{\alpha }\cdot \frac{\partial v_{}^{\bar{\alpha }}}{\partial t}+\rho_{\alpha }\frac{\partial K_{E\alpha }}{\partial t}+\rho_{\alpha }v_{\alpha }\cdot \left(\nabla v_{}^{\bar{\alpha }}\right)\cdot {\tilde{v}}_{\alpha }\\ +\rho_{\alpha }v_{\alpha }\cdot \nabla K_{E\alpha }-{\tilde{v}}_{\alpha }\cdot \left(\nabla \cdot t_{\alpha }\right)-{\tilde{v}}_{\alpha }\cdot \rho_{\alpha }g_{\alpha }=0\;. \end{array}$$

We can average this equation over the α phase within an REV, which yields

(14) $$\begin{array}{cc} {\langle {\tilde{v}}_{\alpha }\cdot \left(P_{\alpha }-v_{\alpha }M_{\alpha }\right)\rangle }_{\Omega_{\alpha },\Omega_{}} & ={\langle \rho_{\alpha }{\tilde{v}}_{\alpha }\cdot \frac{\partial v_{}^{\bar{\alpha }}}{\partial t}\rangle }_{\Omega_{\alpha },\Omega_{}}+{\langle \rho_{\alpha }\frac{\partial K_{E\alpha }}{\partial t}\rangle }_{\Omega_{\alpha },\Omega_{}}\\ & +{\langle \rho_{\alpha }v_{\alpha }\cdot \left(\nabla v_{}^{\bar{\alpha }}\right)\cdot {\tilde{v}}_{\alpha }\rangle }_{\Omega_{\alpha },\Omega_{}}+{\langle \rho_{\alpha }v_{\alpha }\cdot \nabla K_{E\alpha }\rangle }_{\Omega_{\alpha },\Omega_{}}\\ & -{\langle {\tilde{v}}_{\alpha }\cdot \left(\nabla \cdot t_{\alpha }\right)\rangle }_{\Omega_{\alpha },\Omega_{}}-{\langle {\tilde{v}}_{\alpha }\cdot \rho_{\alpha }g_{\alpha }\rangle }_{\Omega_{\alpha },\Omega_{}}=0\;. \end{array}$$

Now, make use of the facts that the density-weighted average of the deviation velocity is zero and that macroscale quantities can be moved outside the averaging operator to simplify this equation to arrive at

(15) $$\begin{array}{cc} {\langle {\tilde{v}}_{\alpha }\cdot \left(P_{\alpha }-v_{\alpha }M_{\alpha }\right)\rangle }_{\Omega_{\alpha },\Omega_{}} & ={\langle \rho_{\alpha }\frac{\partial K_{E\alpha }}{\partial t}\rangle }_{\Omega_{\alpha },\Omega_{}}+{\langle \rho_{\alpha }v_{\alpha }\cdot \nabla K_{E\alpha }\rangle }_{\Omega_{\alpha },\Omega_{}}\\ & +{\langle \rho_{\alpha }{\tilde{v}}_{\alpha }{\tilde{v}}_{\alpha }\rangle }_{\Omega_{\alpha },\Omega_{}}:\nabla v_{}^{\bar{\alpha }}-{\langle {\tilde{v}}_{\alpha }\cdot \left(\nabla \cdot t_{\alpha }\right)\rangle }_{\Omega_{\alpha },\Omega_{}}=0\;. \end{array}$$

Apply the product rule to the first two terms, which gives

(16) $$\begin{array}{cc} {\langle {\tilde{v}}_{\alpha }\cdot \left(P_{\alpha }-v_{\alpha }M_{\alpha }\right)\rangle }_{\Omega_{\alpha },\Omega_{}} & ={\langle \frac{\partial \left(\rho_{\alpha }K_{E\alpha }\right)}{\partial t}\rangle }_{\Omega_{\alpha },\Omega_{}}+{\langle \nabla \cdot (\rho_{\alpha }v_{\alpha }K_{E\alpha })\rangle }_{\Omega_{\alpha },\Omega_{}}\\ & -{\langle K_{E\alpha }\left[\frac{\partial \rho_{\alpha }}{\partial t}+\nabla \cdot \left(\rho_{\alpha }v_{\alpha }\right)\right]\rangle }_{\Omega_{\alpha },\Omega_{}}\\ & +{\langle \rho_{\alpha }{\tilde{v}}_{\alpha }{\tilde{v}}_{\alpha }\rangle }_{\Omega_{\alpha },\Omega_{}}:\nabla v_{}^{\bar{\alpha }}-{\langle {\tilde{v}}_{\alpha }\cdot \left(\nabla \cdot t_{\alpha }\right)\rangle }_{\Omega_{\alpha },\Omega_{}}=0\;. \end{array}$$

The third term on the right-hand side may be eliminated by noting from Equation (6) that the expression in brackets is zero so that

(17) $$\begin{array}{c} {\langle {\tilde{v}}_{\alpha }\cdot \left(P_{\alpha }-v_{\alpha }M_{\alpha }\right)+K_{E\alpha }M_{\alpha }\rangle }_{\Omega_{\alpha },\Omega_{}}=\\ {\langle \frac{\partial \left(\rho_{\alpha }K_{E\alpha }\right)}{\partial t}\rangle }_{\Omega_{\alpha },\Omega_{}}+{\langle \nabla \cdot (\rho_{\alpha }v_{\alpha }K_{E\alpha })\rangle }_{\Omega_{\alpha },\Omega_{}}\\ +{\langle \rho_{\alpha }{\tilde{v}}_{\alpha }{\tilde{v}}_{\alpha }\rangle }_{\Omega_{\alpha },\Omega_{}}:\nabla v_{}^{\bar{\alpha }}-{\langle {\tilde{v}}_{\alpha }\cdot \left(\nabla \cdot t_{\alpha }\right)\rangle }_{\Omega_{\alpha },\Omega_{}}=0\;. \end{array}$$

Next, we take note of the averaging theorems for a phase. First, the average of the divergence of a microscale vector may be related to the divergence of the average of the quantity according to

(18) $${\langle \nabla \cdot f_{\alpha }\rangle }_{\Omega_{\alpha },\Omega_{}}=\nabla \cdot {\langle f_{\alpha }\rangle }_{\Omega_{\alpha },\Omega_{}}+\sum_{\kappa \in I_{c\alpha }^{-}\;}\;{\langle n_{\alpha }\cdot f_{\alpha }\rangle }_{\Omega_{\kappa },\Omega_{}}\;,$$

where $n_{\alpha }$ is the unit vector outward normal from the α phase, and $I_{c\alpha }^{-}$ is the index set of connected entities of one dimension lower than the dimension of the α entity, which would be the set of interfaces formed at the boundary between the α phase and another phases in the system.

The average of a time derivative of a microscale quantity in a phase is related to the time derivative of the average with

(19) $${\langle \frac{\partial f_{\alpha }}{\partial t}\rangle }_{\Omega_{\alpha },\Omega_{}}=\frac{\partial }{\partial t}{\langle f_{\alpha }\rangle }_{\Omega_{\alpha },\Omega_{}}-\sum_{\kappa \in I_{c\alpha }^{-}\;}\;{\langle n_{\alpha }\cdot v_{\kappa }f_{\alpha }\rangle }_{\Omega_{\kappa },\Omega_{}}\;.$$

In Equations (18) and (19), the last terms relate to processes occurring at the boundary of the phase within the averaging volume. Applying these theorems to Equation (17) yields

(20) $$\begin{array}{c} {\langle {\tilde{v}}_{\alpha }\cdot \left(P_{\alpha }-v_{\alpha }M_{\alpha }\right)+K_{E\alpha }M_{\alpha }\rangle }_{\Omega_{\alpha },\Omega_{}}=\\ \frac{\partial }{\partial t}{\langle \rho_{\alpha }K_{E\alpha }\rangle }_{\Omega_{\alpha },\Omega_{}}+\nabla \cdot {\langle \rho_{\alpha }v_{\alpha }K_{E\alpha }\rangle }_{\Omega_{\alpha },\Omega_{}}\\ +\sum_{\kappa \in I_{c\alpha }^{-}\;}\;{\langle n_{\alpha }\cdot \rho_{\alpha }\left(v_{\alpha }-v_{\kappa }\right)K_{E\alpha }\rangle }_{\Omega_{\kappa },\Omega_{}}\\ +{\langle \rho_{\alpha }{\tilde{v}}_{\alpha }{\tilde{v}}_{\alpha }\rangle }_{\Omega_{\alpha },\Omega_{}}:\nabla v_{}^{\bar{\alpha }}-{\langle {\tilde{v}}_{\alpha }\cdot \left(\nabla \cdot t_{\alpha }\right)\rangle }_{\Omega_{\alpha },\Omega_{}}=0\;. \end{array}$$

Note that the third line of Equation (20) contains a difference between the velocity of a phase and a velocity of an interface evaluated on the interface. This term will vanish for the case in which no mass exchange between the phase and the interface occurs at the microscale.

Arranging Equation (20) further, making use of the definition of the average of the kinetic energy term and the definition of the mass exchange from the κ interface to the α phase,

(21) $$\underset{\kappa \to \alpha }{M}:=-n_{\alpha }\cdot \rho_{\alpha }\left(v_{\alpha }-v_{\kappa }\right)\;\mathrm{for}\;\kappa \in I_{c\alpha }^{-}\;,$$

and the expression for the momentum transfer from the κ interface to the α phase,

(22) $$\underset{\kappa \to \alpha }{T}:=-n_{\alpha }\cdot t_{\alpha }\;\mathrm{for}\;\kappa \in I_{c\alpha }^{-}\;,$$

to obtain

(23) $$\begin{array}{c} {\langle {\tilde{v}}_{\alpha }\cdot \left(P_{\alpha }-v_{\alpha }M_{\alpha }\right)+K_{E\alpha }M_{\alpha }\rangle }_{\Omega_{\alpha },\Omega_{}}=\\ \frac{\partial \left(\epsilon^{\bar{\bar{\alpha }}}\rho^{\alpha }K_{E}^{\bar{\bar{\alpha }}}\right)}{\partial t}+\nabla \cdot \left(\epsilon^{\bar{\bar{\alpha }}}\rho^{\alpha }K_{E}^{\bar{\bar{\alpha }}}v_{}^{\bar{\alpha }}\right)+\nabla \cdot {\langle \left[\rho_{\alpha }{\tilde{v}}_{\alpha }K_{E\alpha }-t_{\alpha }\cdot {\tilde{v}}_{\alpha }\right]\rangle }_{\Omega_{\alpha },\Omega_{}}\\ -\sum_{\kappa \in I_{c\alpha }^{-}\;}\;{\langle \left[\underset{\kappa \to \alpha }{M}K_{E\alpha }+\underset{\kappa \to \alpha }{T}\cdot {\tilde{v}}_{\alpha }\right]\rangle }_{\Omega_{\kappa },\Omega_{}}\\ +{\langle \rho_{\alpha }{\tilde{v}}_{\alpha }{\tilde{v}}_{\alpha }\rangle }_{\Omega_{\alpha },\Omega_{}}:d^{\bar{\bar{\alpha }}}+{\langle t_{\alpha }:\nabla {\tilde{v}}_{\alpha }\rangle }_{\Omega_{\alpha },\Omega_{}}=0\;, \end{array}$$

where $d^{\bar{\bar{\alpha }}}$ is the rate of strain tensor.

Apply the product rule to the first two terms on the right side of Equation (23) and collect terms which gives

(24) $$\begin{array}{cc} K_{*1}^{\bar{\bar{\alpha }}} & ={\langle {\tilde{v}}_{\alpha }\cdot \left(P_{\alpha }-v_{\alpha }M_{\alpha }\right)+K_{E\alpha }M_{\alpha }\rangle }_{\Omega_{\alpha },\Omega_{}}=\\ & \epsilon^{\bar{\bar{\alpha }}}\rho^{\alpha }\frac{D^{\bar{\alpha }}K_{E}^{\bar{\bar{\alpha }}}}{Dt}+K_{E}^{\bar{\bar{\alpha }}}\frac{D^{\bar{\alpha }}\left(\epsilon^{\bar{\bar{\alpha }}}\rho^{\alpha }\right)}{Dt}+\left(\epsilon^{\bar{\bar{\alpha }}}\rho^{\alpha }K_{E}^{\bar{\bar{\alpha }}}I+{\langle \rho_{\alpha }{\tilde{v}}_{\alpha }{\tilde{v}}_{\alpha }\rangle }_{\Omega_{\alpha },\Omega_{}}\right):d^{\bar{\bar{\alpha }}}+\nabla \cdot {\langle \left[\rho_{\alpha }{\tilde{v}}_{\alpha }K_{E\alpha }-t_{\alpha }\cdot {\tilde{v}}_{\alpha }\right]\rangle }_{\Omega_{\alpha },\Omega_{}}\\ & -\sum_{\kappa \in I_{c\alpha }^{-}\;}\;{\langle \left[\underset{\kappa \to \alpha }{M}K_{E\alpha }+\underset{\kappa \to \alpha }{T}\cdot {\tilde{v}}_{\alpha }\right]\rangle }_{\Omega_{\kappa },\Omega_{}}+{\langle t_{\alpha }:\nabla {\tilde{v}}_{\alpha }\rangle }_{\Omega_{\alpha },\Omega_{}}=0\;. \end{array}$$

As an alternative, subtract $K_{E}^{\bar{\bar{\alpha }}}M_{*}^{\bar{\bar{\alpha }}}$ from this equation to yield:

(25) $$\begin{array}{cc} K_{*2}^{\bar{\bar{\alpha }}} & ={\langle {\tilde{v}}_{\alpha }\cdot \left(P_{\alpha }-v_{\alpha }M_{\alpha }\right)+K_{E\alpha }M_{\alpha }\rangle }_{\Omega_{\alpha },\Omega_{}}-K_{E}^{\bar{\bar{\alpha }}}M_{*}^{\bar{\bar{\alpha }}}=\\ & \epsilon^{\bar{\bar{\alpha }}}\rho^{\alpha }\frac{D^{\bar{\alpha }}K_{E}^{\bar{\bar{\alpha }}}}{Dt}+{\langle \rho_{\alpha }{\tilde{v}}_{\alpha }{\tilde{v}}_{\alpha }\rangle }_{\Omega_{\alpha },\Omega_{}}:d^{\bar{\bar{\alpha }}}+\nabla \cdot {\langle \left[\rho_{\alpha }{\tilde{v}}_{\alpha }K_{E\alpha }-t_{\alpha }\cdot {\tilde{v}}_{\alpha }\right]\rangle }_{\Omega_{\alpha },\Omega_{}}\\ & -\sum_{\kappa \in I_{c\alpha }^{-}\;}\;{\langle \left[\underset{\kappa \to \alpha }{M}\left(K_{E\alpha }-K_{E}^{\bar{\bar{\alpha }}}\right)+\underset{\kappa \to \alpha }{T}\cdot {\tilde{v}}_{\alpha }\right]\rangle }_{\Omega_{\kappa },\Omega_{}}+{\langle t_{\alpha }:\nabla {\tilde{v}}_{\alpha }\rangle }_{\Omega_{\alpha },\Omega_{}}=0\;. \end{array}$$

Note that Equation (25) is related to Equation (24) by

(26) $$K_{*2}^{\bar{\bar{\alpha }}}=K_{*1}^{\bar{\bar{\alpha }}}-K_{E}^{\bar{\bar{\alpha }}}M_{*}^{\bar{\bar{\alpha }}}\;.$$

Whether $K_{*2}^{\bar{\bar{\alpha }}}$ or $K_{*1}^{\bar{\bar{\alpha }}}$ is used in constraining the entropy inequality does not really matter as the difference between the two will be accounted for by the Lagrange multipliers.

### 4.3. Macroscale Deviation Kinetic Energy for an Interface

Equation (25) provides the deviation kinetic energy for a phase explicitly in terms of averaged quantities but also in terms of general expressions for macroscale equations. The corresponding deviation kinetic energy equation for an interface will be different because the interface is in ${I\;R}^{2}$ rather than ${I\;R}^{3}$. We expect that the equation will involve the same equations for an interface as were used for a phase such that

(27) $$K_{*2}^{\bar{\bar{\alpha }}}={\langle {\tilde{v}}_{\alpha }\cdot \left(P_{\alpha }-v_{\alpha }M_{\alpha }\right)+K_{E\alpha }M_{\alpha }\rangle }_{\Omega_{\alpha },\Omega_{}}-K_{E}^{\bar{\bar{\alpha }}}M_{*}^{\bar{\bar{\alpha }}}\;\mathrm{for}\;\alpha \in I_{I}\;,$$

where $I_{I}$ is the index set of interfaces in the system.

The TCAT averaging operator for an interface is a normalized weighted integration over all of the interfaces of a given type in the REV, or explicitly for a density-weighted velocity of an interface as

(28) $$v_{}^{\bar{\alpha }}={\langle v_{\alpha }\rangle }_{\Omega_{\alpha },\Omega_{\alpha },\rho_{\alpha }}=\frac{\int_{\Omega_{\alpha }}\rho_{\alpha }v_{\alpha }\;dr}{\int_{\Omega_{\alpha }}\rho_{\alpha }\;dr}\;\mathrm{for}\;\alpha \in I_{I}\;.$$

It is also important to note that at the microscale the differential operators must be restricted to a two-dimensional form such that a microscale point remains on the potentially moving interface.

The microscale conservation of mass equation for an interface is

(29) $$M_{\alpha }=\frac{\partial^{\prime }\rho_{\alpha }}{\partial t}+\nabla^{\prime }\cdot \left(\rho_{\alpha }v_{\alpha }\right)-\sum_{\kappa \in I_{c\alpha }^{+}\;}\;\underset{\kappa \to \alpha }{M}=0\;\mathrm{for}\;\alpha \in I_{I}\;,$$

where $I_{c\alpha }^{+}$ is the index set of phases that form interface α, and the microscale expression for conservation of interface momentum is

(30) $$\begin{array}{cc} P_{\alpha }= & \frac{\partial^{\prime }\left(\rho_{\alpha }v_{\alpha }\right)}{\partial t}+\nabla^{\prime }\cdot \left(\rho_{\alpha }v_{\alpha }v_{\alpha }\right)-\nabla^{\prime }\cdot \left(I^{\prime }\cdot t_{\alpha }\right)-\rho_{\alpha }g_{\alpha }\\ & -\sum_{\kappa \in I_{c\alpha }^{+}\;}\;\left(v_{\kappa }\underset{\kappa \to \alpha }{M}+\underset{\kappa \to \alpha }{T}\right)=0\;\mathrm{for}\;\alpha \in I_{I}\;. \end{array}$$

In these equations, the time derivative and divergence operator are indicated as being restricted to a surface by the prime. Additionally the mass exchange term is

(31) $$\underset{\kappa \to \alpha }{M}:=-n_{\kappa }\cdot \left[\rho_{\kappa }\left(v_{\alpha }-v_{\kappa }\right)\right]\;\mathrm{for}\;\kappa \in I_{c\alpha }^{+}\;,$$

and the expression for the momentum transfer from the κ interface to the α phase is

(32) $$\underset{\kappa \to \alpha }{T}:=-n_{\kappa }\cdot t_{\kappa }\;\mathrm{for}\;\kappa \in I_{c\alpha }^{+}\;.$$

In light of Equation (27), we combine Equations (30) and (29) to obtain

(33) $$\begin{array}{cc} P_{\alpha }-v_{\alpha }M_{\alpha } & =\rho_{\alpha }\frac{\partial^{\prime }v_{\alpha }}{\partial t}+\rho_{\alpha }v_{\alpha }\cdot \nabla^{\prime }v_{\alpha }-\nabla^{\prime }\cdot \left(I^{\prime }\cdot t_{\alpha }\right)-\rho_{\alpha }g_{\alpha }\\ & -\sum_{\kappa \in I_{c\alpha }^{+}\;}\;\left[\left(v_{\kappa }-v_{\alpha }\right)\underset{\kappa \to \alpha }{M}+\underset{\kappa \to \alpha }{T}\right]=0\;. \end{array}$$

Introduction of Equation (4) into this expression yields

(34) $$\begin{array}{cc} \rho_{\alpha }\frac{\partial^{\prime }\left(v_{}^{\bar{\alpha }}+{\tilde{v}}_{\alpha }\right)}{\partial t} & +\rho_{\alpha }v_{\alpha }\cdot \nabla^{\prime }\left(v_{}^{\bar{\alpha }}+{\tilde{v}}_{\alpha }\right)-\nabla^{\prime }\cdot \left(I^{\prime }\cdot t_{\alpha }\right)-\rho_{\alpha }g_{\alpha }\\ & -\sum_{\kappa \in I_{c\alpha }^{+}\;}\;\left[\left(v_{\kappa }-v_{\alpha }\right)\underset{\kappa \to \alpha }{M}+\underset{\kappa \to \alpha }{T}\right]=0\;. \end{array}$$

Taking the dot product of Equation (34) with ${\tilde{v}}_{\alpha }$, expanding terms, and applying the product rule yields

(35) $$\begin{array}{c} {\tilde{v}}_{\alpha }\cdot \left(P_{\alpha }-v_{\alpha }M_{\alpha }\right)=\rho_{\alpha }{\tilde{v}}_{\alpha }\cdot \frac{\partial^{\prime }v_{}^{\bar{\alpha }}}{\partial t}+\rho_{\alpha }\frac{\partial^{\prime }}{\partial t}\left(\frac{{\tilde{v}}_{\alpha }\cdot {\tilde{v}}_{\alpha }}{2}\right)+\rho_{\alpha }v_{\alpha }\cdot \left(\nabla^{\prime }v_{}^{\bar{\alpha }}\right)\cdot {\tilde{v}}_{\alpha }\\ +\rho_{\alpha }v_{\alpha }\cdot \nabla^{\prime }\left(\frac{{\tilde{v}}_{\alpha }\cdot {\tilde{v}}_{\alpha }}{2}\right)-\nabla^{\prime }\cdot \left(I^{\prime }\cdot t_{\alpha }\right)\cdot {\tilde{v}}_{\alpha }-\rho_{\alpha }{\tilde{v}}_{\alpha }\cdot g_{\alpha }\\ -\sum_{\kappa \in I_{c\alpha }^{+}\;}\;{\tilde{v}}_{\alpha }\cdot \left[\left(v_{\kappa }-v_{\alpha }\right)\underset{\kappa \to \alpha }{M}+\underset{\kappa \to \alpha }{T}\right]=0\;. \end{array}$$

This may be written in terms of the microscale kinetic energy deviation as:

(36) $$\begin{array}{c} {\tilde{v}}_{\alpha }\cdot \left(P_{\alpha }-v_{\alpha }M_{\alpha }\right)=\rho_{\alpha }{\tilde{v}}_{\alpha }\cdot \frac{\partial^{\prime }v_{}^{\bar{\alpha }}}{\partial t}+\rho_{\alpha }\frac{\partial^{\prime }K_{E\alpha }}{\partial t}+\rho_{\alpha }v_{\alpha }\cdot \left(\nabla^{\prime }v_{}^{\bar{\alpha }}\right)\cdot {\tilde{v}}_{\alpha }\\ +\rho_{\alpha }v_{\alpha }\cdot \nabla^{\prime }K_{E\alpha }-\nabla^{\prime }\cdot \left(I^{\prime }\cdot t_{\alpha }\right)\cdot {\tilde{v}}_{\alpha }-\rho_{\alpha }{\tilde{v}}_{\alpha }\cdot g_{\alpha }\\ -\sum_{\kappa \in I_{c\alpha }^{+}\;}\;{\tilde{v}}_{\alpha }\cdot \left[\left(v_{\kappa }-v_{\alpha }\right)\underset{\kappa \to \alpha }{M}+\underset{\kappa \to \alpha }{T}\right]=0\;. \end{array}$$

Here, we make use of the relation:

(37) $$\frac{\partial^{\prime }}{\partial t}+v_{\alpha }\cdot \nabla^{\prime }=\frac{\partial }{\partial t}+v_{\alpha }\cdot \nabla$$

to rewrite Equation (36) as:

(38) $$\begin{array}{c} {\tilde{v}}_{\alpha }\cdot \left(P_{\alpha }-v_{\alpha }M_{\alpha }\right)=\rho_{\alpha }{\tilde{v}}_{\alpha }\cdot \frac{\partial v_{}^{\bar{\alpha }}}{\partial t}+\rho_{\alpha }\frac{\partial^{\prime }K_{E\alpha }}{\partial t}+\rho_{\alpha }v_{\alpha }\cdot \left(\nabla v_{}^{\bar{\alpha }}\right)\cdot {\tilde{v}}_{\alpha }\\ +\rho_{\alpha }v_{\alpha }\cdot \nabla^{\prime }K_{E\alpha }-\nabla^{\prime }\cdot \left(I^{\prime }\cdot t_{\alpha }\right)\cdot {\tilde{v}}_{\alpha }-\rho_{\alpha }{\tilde{v}}_{\alpha }\cdot g_{\alpha }\\ -\sum_{\kappa \in I_{c\alpha }^{+}\;}\;{\tilde{v}}_{\alpha }\cdot \left[\left(v_{\kappa }-v_{\alpha }\right)\underset{\kappa \to \alpha }{M}+\underset{\kappa \to \alpha }{T}\right]=0\;. \end{array}$$

Take the average of this equation and move macroscale quantities outside the operator while dropping terms that average to zero. The result is

(39) $$\begin{array}{c} {\langle {\tilde{v}}_{\alpha }\cdot \left(P_{\alpha }-v_{\alpha }M_{\alpha }\right)\rangle }_{\Omega_{\alpha },\Omega_{}}={\langle \rho_{\alpha }\frac{\partial^{\prime }K_{E\alpha }}{\partial t}\rangle }_{\Omega_{\alpha },\Omega_{}}+{\langle \rho_{\alpha }v_{\alpha }\cdot \nabla^{\prime }K_{E\alpha }\rangle }_{\Omega_{\alpha },\Omega_{}}\\ +{\langle \rho_{\alpha }{\tilde{v}}_{\alpha }{\tilde{v}}_{\alpha }\rangle }_{\Omega_{\alpha },\Omega_{}}:d^{\bar{\bar{\alpha }}}-{\langle \nabla^{\prime }\cdot \left(I^{\prime }\cdot t_{\alpha }\right)\cdot {\tilde{v}}_{\alpha }\rangle }_{\Omega_{\alpha },\Omega_{}}\\ -\sum_{\kappa \in I_{c\alpha }^{+}\;}\;{\langle {\tilde{v}}_{\alpha }\cdot \left[\left(v_{\kappa }-v_{\alpha }\right)\underset{\kappa \to \alpha }{M}+\underset{\kappa \to \alpha }{T}\right]\rangle }_{\Omega_{\alpha },\Omega_{}}=0\;. \end{array}$$

Apply the product rule to the first, second, and fourth terms in this equation to obtain

(40) $$\begin{array}{c} {\langle {\tilde{v}}_{\alpha }\cdot \left(P_{\alpha }-v_{\alpha }M_{\alpha }\right)\rangle }_{\Omega_{\alpha },\Omega_{}}={\langle \frac{\partial^{\prime }\left(\rho_{\alpha }K_{E\alpha }\right)}{\partial t}\rangle }_{\Omega_{\alpha },\Omega_{}}+{\langle \nabla^{\prime }\cdot \left(\rho_{\alpha }v_{\alpha }K_{E\alpha }\right)\rangle }_{\Omega_{\alpha },\Omega_{}}\\ -{\langle K_{E\alpha }\left[\frac{\partial^{\prime }\rho_{\alpha }}{\partial t}+\nabla^{\prime }\cdot \left(\rho_{\alpha }v_{\alpha }\right)\right]\rangle }_{\Omega_{\alpha },\Omega_{}}\\ +{\langle \rho_{\alpha }{\tilde{v}}_{\alpha }{\tilde{v}}_{\alpha }\rangle }_{\Omega_{\alpha },\Omega_{}}:d^{\bar{\bar{\alpha }}}-{\langle \nabla^{\prime }\cdot \left(I^{\prime }\cdot t_{\alpha }\cdot {\tilde{v}}_{\alpha }\right)\rangle }_{\Omega_{\alpha },\Omega_{}}+{\langle I^{\prime }\cdot t_{\alpha }:{\left(\nabla^{\prime }{\tilde{v}}_{\alpha }\right)}^{T}\rangle }_{\Omega_{\alpha },\Omega_{}}\\ -\sum_{\kappa \in I_{c\alpha }^{+}\;}\;{\langle {\tilde{v}}_{\alpha }\cdot \left[\left(v_{\kappa }-v_{\alpha }\right)\underset{\kappa \to \alpha }{M}+\underset{\kappa \to \alpha }{T}\right]\rangle }_{\Omega_{\alpha },\Omega_{}}=0\;. \end{array}$$

Add to this expression the quantity ${\langle K_{E\alpha }M_{\alpha }\rangle }_{\Omega_{\alpha },\Omega_{}}$ so that we have

(41) $$\begin{array}{c} {\langle {\tilde{v}}_{\alpha }\cdot \left(P_{\alpha }-v_{\alpha }M_{\alpha }\right)+K_{E\alpha }M_{\alpha }\rangle }_{\Omega_{\alpha },\Omega_{}}=\\ {\langle \frac{\partial^{\prime }\left(\rho_{\alpha }K_{E\alpha }\right)}{\partial t}\rangle }_{\Omega_{\alpha },\Omega_{}}+{\langle \nabla^{\prime }\cdot \left(\rho_{\alpha }v_{\alpha }K_{E\alpha }\right)\rangle }_{\Omega_{\alpha },\Omega_{}}\\ -{\langle \sum_{\kappa \in I_{c\alpha }^{+}\;}\;K_{E\alpha }\underset{\kappa \to \alpha }{M}\rangle }_{\Omega_{\alpha },\Omega_{}}+{\langle \rho_{\alpha }{\tilde{v}}_{\alpha }{\tilde{v}}_{\alpha }\rangle }_{\Omega_{\alpha },\Omega_{}}:d^{\bar{\bar{\alpha }}}\\ -{\langle \nabla^{\prime }\cdot \left(I^{\prime }\cdot t_{\alpha }\cdot {\tilde{v}}_{\alpha }\right)\rangle }_{\Omega_{\alpha },\Omega_{}}+{\langle I^{\prime }\cdot t_{\alpha }:{\left(\nabla^{\prime }{\tilde{v}}_{\alpha }\right)}^{T}\rangle }_{\Omega_{\alpha },\Omega_{}}\\ -\sum_{\kappa \in I_{c\alpha }^{+}\;}\;{\langle {\tilde{v}}_{\alpha }\cdot \left[\left(v_{\kappa }-v_{\alpha }\right)\underset{\kappa \to \alpha }{M}+\underset{\kappa \to \alpha }{T}\right]\rangle }_{\Omega_{\alpha },\Omega_{}}=0\;. \end{array}$$

Next, we make use of the averaging theorems for surfaces. For a surface divergence, the relation is

(42) $${\langle \nabla^{\prime }\cdot f_{\alpha }\rangle }_{\Omega_{\alpha },\Omega_{}}=\nabla \cdot {\langle I_{\alpha }^{\prime }\cdot f_{\alpha }\rangle }_{\Omega_{\alpha },\Omega_{}}-{\langle \nabla^{\prime }\cdot I_{\alpha }^{\prime }\cdot f_{\alpha }\rangle }_{\Omega_{\alpha },\Omega_{}}+\sum_{\kappa \in I_{c\alpha }^{-}\;}\;{\langle n_{\alpha }\cdot f_{\alpha }\rangle }_{\Omega_{\kappa },\Omega_{}}\;,$$

while the expression for the partial time derivative constrained to the surface is

(43) $$\begin{array}{c} {\langle \frac{\partial^{\prime }f_{\alpha }}{\partial t}\rangle }_{\Omega_{\alpha },\Omega_{}}=\frac{\partial }{\partial t}{\langle f_{\alpha }\rangle }_{\Omega_{\alpha },\Omega_{}}+\nabla \cdot {\langle \left(I-I_{\alpha }^{\prime }\right)\cdot v_{\alpha }f_{\alpha }\rangle }_{\Omega_{\alpha },\Omega_{}}\\ +{\langle \nabla^{\prime }\cdot I_{\alpha }^{\prime }\cdot v_{\alpha }f_{\alpha }\rangle }_{\Omega_{\alpha },\Omega_{}}-\sum_{\kappa \in I_{c\alpha }^{-}\;}\;{\langle n_{\alpha }\cdot v_{\kappa }f_{\alpha }\rangle }_{\Omega_{\kappa },\Omega_{}}\;. \end{array}$$

Apply these to the first two terms after the equal sign in Equation (41) and to the divergence of the operator in the fifth term so that we obtain, after recombining terms,

(44) $$\begin{array}{c} {\langle {\tilde{v}}_{\alpha }\cdot \left(P_{\alpha }-v_{\alpha }M_{\alpha }\right)+K_{E\alpha }M_{\alpha }\rangle }_{\Omega_{\alpha },\Omega_{}}=\\ \epsilon^{\bar{\bar{\alpha }}}\rho^{\alpha }\frac{D^{\bar{\alpha }}K_{E}^{\bar{\bar{\alpha }}}}{Dt}+K_{E}^{\bar{\bar{\alpha }}}\frac{D^{\bar{\alpha }}\left(\epsilon^{\bar{\bar{\alpha }}}\rho^{\alpha }\right)}{Dt}+\left(\epsilon^{\bar{\bar{\alpha }}}\rho^{\alpha }K_{E}^{\bar{\bar{\alpha }}}I+{\langle \rho_{\alpha }{\tilde{v}}_{\alpha }{\tilde{v}}_{\alpha }\rangle }_{\Omega_{\alpha },\Omega_{}}\right):d^{\bar{\bar{\alpha }}}\\ +\nabla \cdot {\langle \left(\rho_{\alpha }K_{E\alpha }{\tilde{v}}_{\alpha }-I^{\prime }\cdot t_{\alpha }\cdot {\tilde{v}}_{\alpha }\right)\rangle }_{\Omega_{\alpha },\Omega_{}}\\ +\sum_{\kappa \in I_{c\alpha }^{-}\;}\;{\langle n_{\alpha }\cdot \left[\rho_{\alpha }\left(v_{\alpha }-v_{\kappa }\right)K_{E\alpha }-t_{\alpha }\cdot {\tilde{v}}_{\alpha }\right]\rangle }_{\Omega_{\kappa },\Omega_{}}\\ -\sum_{\kappa \in I_{c\alpha }^{+}\;}\;{\langle \left[{\tilde{v}}_{\alpha }\cdot \left(v_{\kappa }-v_{\alpha }\right)+K_{E\alpha }\right]\underset{\kappa \to \alpha }{M}+{\tilde{v}}_{\alpha }\cdot \underset{\kappa \to \alpha }{T}\rangle }_{\Omega_{\alpha },\Omega_{}}\\ +{\langle I^{\prime }\cdot t_{\alpha }:{\left(\nabla^{\prime }{\tilde{v}}_{\alpha }\right)}^{T}\rangle }_{\Omega_{\alpha },\Omega_{}}=0\;. \end{array}$$

Make use of the definitions for the exchange terms between entities given by Equations (21), (31), and (32) to simplify the notation to

(45) $$\begin{array}{c} K_{*1}^{\bar{\bar{\alpha }}}={\langle {\tilde{v}}_{\alpha }\cdot \left(P_{\alpha }-v_{\alpha }M_{\alpha }\right)+K_{E\alpha }M_{\alpha }\rangle }_{\Omega_{\alpha },\Omega_{}}=\\ \epsilon^{\bar{\bar{\alpha }}}\rho^{\alpha }\frac{D^{\bar{\alpha }}K_{E}^{\bar{\bar{\alpha }}}}{Dt}+K_{E}^{\bar{\bar{\alpha }}}\frac{D^{\bar{\alpha }}\left(\epsilon^{\bar{\bar{\alpha }}}\rho^{\alpha }\right)}{Dt}+\left(\epsilon^{\bar{\bar{\alpha }}}\rho^{\alpha }K_{E}^{\bar{\bar{\alpha }}}I+{\langle \rho_{\alpha }{\tilde{v}}_{\alpha }{\tilde{v}}_{\alpha }\rangle }_{\Omega_{\alpha },\Omega_{}}\right):d^{\bar{\bar{\alpha }}}\\ +\nabla \cdot {\langle \rho_{\alpha }K_{E\alpha }{\tilde{v}}_{\alpha }-I^{\prime }\cdot t_{\alpha }\cdot {\tilde{v}}_{\alpha }\rangle }_{\Omega_{\alpha },\Omega_{}}\\ -\sum_{\kappa \in I_{c\alpha }^{-}\;}\;{\langle \underset{\kappa \to \alpha }{M}K_{E\alpha }+\underset{\kappa \to \alpha }{T}\cdot {\tilde{v}}_{\alpha }\rangle }_{\Omega_{\kappa },\Omega_{}}\\ -\sum_{\kappa \in I_{c\alpha }^{+}\;}\;{\langle \underset{\kappa \to \alpha }{M}K_{E\alpha }+\left[\underset{\kappa \to \alpha }{T}+\left(v_{\kappa }-v_{\alpha }\right)\underset{\kappa \to \alpha }{M}\right]\cdot {\tilde{v}}_{\alpha }\rangle }_{\Omega_{\alpha },\Omega_{}}\\ +{\langle I^{\prime }\cdot t_{\alpha }:{\left(\nabla^{\prime }{\tilde{v}}_{\alpha }\right)}^{T}\rangle }_{\Omega_{\alpha },\Omega_{}}=0\;\mathrm{for}\;\alpha \in I_{I}\;. \end{array}$$

We also obtain

(46) $$\begin{array}{c} K_{*2}^{\bar{\bar{\alpha }}}={\langle {\tilde{v}}_{\alpha }\cdot \left(P_{\alpha }-v_{\alpha }M_{\alpha }\right)+K_{E\alpha }M_{\alpha }\rangle }_{\Omega_{\alpha },\Omega_{}}-K_{E}^{\bar{\bar{\alpha }}}M_{*}^{\bar{\bar{\alpha }}}=\\ \epsilon^{\bar{\bar{\alpha }}}\rho^{\alpha }\frac{D^{\bar{\alpha }}K_{E}^{\bar{\bar{\alpha }}}}{Dt}+{\langle \rho_{\alpha }{\tilde{v}}_{\alpha }{\tilde{v}}_{\alpha }\rangle }_{\Omega_{\alpha },\Omega_{}}:d^{\bar{\bar{\alpha }}}\\ +\nabla \cdot {\langle \rho_{\alpha }K_{E\alpha }{\tilde{v}}_{\alpha }-I^{\prime }\cdot t_{\alpha }\cdot {\tilde{v}}_{\alpha }\rangle }_{\Omega_{\alpha },\Omega_{}}\\ -\sum_{\kappa \in I_{c\alpha }^{-}\;}\;{\langle \underset{\kappa \to \alpha }{M}\left(K_{E\alpha }-K_{E}^{\bar{\bar{\alpha }}}\right)+\underset{\kappa \to \alpha }{T}\cdot {\tilde{v}}_{\alpha }\rangle }_{\Omega_{\kappa },\Omega_{}}\\ -\sum_{\kappa \in I_{c\alpha }^{+}\;}\;{\langle \underset{\kappa \to \alpha }{M}\left(K_{E\alpha }-K_{E}^{\bar{\bar{\alpha }}}\right)+\left[\underset{\kappa \to \alpha }{T}+\left(v_{\kappa }-v_{\alpha }\right)\underset{\kappa \to \alpha }{M}\right]\cdot {\tilde{v}}_{\alpha }\rangle }_{\Omega_{\alpha },\Omega_{}}\\ +{\langle I^{\prime }\cdot t_{\alpha }:{\left(\nabla^{\prime }{\tilde{v}}_{\alpha }\right)}^{T}\rangle }_{\Omega_{\alpha },\Omega_{}}=0\;\mathrm{for}\;\alpha \in I_{I}\;. \end{array}$$

### 4.4. Macroscale Deviation Kinetic Energy for a Common Curve

The difference between the expressions for $K_{*1}^{\bar{\bar{\alpha }}}$ and $K_{*2}^{\bar{\bar{\alpha }}}$ for phases and the corresponding expressions for interfaces are in the presence of interactions with a higher dimensional entity and the stress tensor being in the appropriate space. Based on these observations, we can write the expressions for the common curve as

(47) $$\begin{array}{c} K_{*1}^{\bar{\bar{\alpha }}}={\langle {\tilde{v}}_{\alpha }\cdot \left(P_{\alpha }-v_{\alpha }M_{\alpha }\right)+K_{E\alpha }M_{\alpha }\rangle }_{\Omega_{\alpha },\Omega_{}}=\\ \epsilon^{\bar{\bar{\alpha }}}\rho^{\alpha }\frac{D^{\bar{\alpha }}K_{E}^{\bar{\bar{\alpha }}}}{Dt}+K_{E}^{\bar{\bar{\alpha }}}\frac{D^{\bar{\alpha }}\left(\epsilon^{\bar{\bar{\alpha }}}\rho^{\alpha }\right)}{Dt}+\left(\epsilon^{\bar{\bar{\alpha }}}\rho^{\alpha }K_{E}^{\bar{\bar{\alpha }}}I+{\langle \rho_{\alpha }{\tilde{v}}_{\alpha }{\tilde{v}}_{\alpha }\rangle }_{\Omega_{\alpha },\Omega_{}}\right):d^{\bar{\bar{\alpha }}}\\ +\nabla \cdot {\langle \rho_{\alpha }K_{E\alpha }{\tilde{v}}_{\alpha }-I^{\prime \prime }\cdot t_{\alpha }\cdot {\tilde{v}}_{\alpha }\rangle }_{\Omega_{\alpha },\Omega_{}}\\ -\sum_{\kappa \in I_{c\alpha }^{+}\;}\;{\langle \underset{\kappa \to \alpha }{M}K_{E\alpha }+\left[\underset{\kappa \to \alpha }{T}+\left(v_{\kappa }-v_{\alpha }\right)\underset{\kappa \to \alpha }{M}\right]\cdot {\tilde{v}}_{\alpha }\rangle }_{\Omega_{\alpha },\Omega_{}}\\ +{\langle I^{\prime \prime }\cdot t_{\alpha }:{\left(\nabla^{\prime \prime }{\tilde{v}}_{\alpha }\right)}^{T}\rangle }_{\Omega_{\alpha },\Omega_{}}=0\;\mathrm{for}\;\alpha \in I_{C}\;. \end{array}$$

We also obtain

(48) $$\begin{array}{c} K_{*2}^{\bar{\bar{\alpha }}}={\langle {\tilde{v}}_{\alpha }\cdot \left(P_{\alpha }-v_{\alpha }M_{\alpha }\right)+K_{E\alpha }M_{\alpha }\rangle }_{\Omega_{\alpha },\Omega_{}}-K_{E}^{\bar{\bar{\alpha }}}M_{*}^{\bar{\bar{\alpha }}}=\\ \epsilon^{\bar{\bar{\alpha }}}\rho^{\alpha }\frac{D^{\bar{\alpha }}K_{E}^{\bar{\bar{\alpha }}}}{Dt}+{\langle \rho_{\alpha }{\tilde{v}}_{\alpha }{\tilde{v}}_{\alpha }\rangle }_{\Omega_{\alpha },\Omega_{}}:d^{\bar{\bar{\alpha }}}\\ +\nabla \cdot {\langle \rho_{\alpha }K_{E\alpha }{\tilde{v}}_{\alpha }-I^{\prime \prime }\cdot t_{\alpha }\cdot {\tilde{v}}_{\alpha }\rangle }_{\Omega_{\alpha },\Omega_{}}\\ -\sum_{\kappa \in I_{c\alpha }^{+}\;}\;{\langle \underset{\kappa \to \alpha }{M}\left(K_{E\alpha }-K_{E}^{\bar{\bar{\alpha }}}\right)+\left[\underset{\kappa \to \alpha }{T}+\left(v_{\kappa }-v_{\alpha }\right)\underset{\kappa \to \alpha }{M}\right]\cdot {\tilde{v}}_{\alpha }\rangle }_{\Omega_{\alpha },\Omega_{}}\\ +{\langle I^{\prime \prime }\cdot t_{\alpha }:{\left(\nabla^{\prime \prime }{\tilde{v}}_{\alpha }\right)}^{T}\rangle }_{\Omega_{\alpha },\Omega_{}}=0\;\mathrm{for}\;\alpha \in I_{C}\;, \end{array}$$

where the double prime denotes restriction to the one-dimensional common curve. In these equations, there is no exchange with a lower dimensional entity, which would be a common point, since common points are excluded in this system.

## 5. Formulation of CEI

In the formulation of a CEI for porous media analyses, the macroscale entropy balance for each entity ($S_{*}^{\bar{\bar{\alpha }}}$) is summed over all entities. This equation is then constrained using Lagrange multipliers that multiply the macroscale conservation equations for each entity consisting of the total energy equation ($E_{*}^{\bar{\bar{\alpha }}}$), the momentum equation ($P_{*}^{\alpha }$), and the mass conservation equation ($M_{*}^{\bar{\bar{\alpha }}}$). Additional constraints are provided by the macroscale relation between the body force potential for a phase and body force ($G_{*}^{\bar{\bar{\alpha }}}$), the thermodynamic formalism ($T_{*}^{\bar{\bar{\alpha }}}$), and an expression for the material derivative of the body force potential ($T_{G*}^{\bar{\bar{\alpha }}}$). Thus, the CEI, with Lagrange multipliers indicated as λ coefficients, has previously been written as

(49) $$\begin{array}{c} \sum_{\alpha \in I\;}\;S_{*}^{\bar{\bar{\alpha }}}+\sum_{\alpha \in I\;}\;\lambda_{E}^{\alpha }E_{*}^{\bar{\bar{\alpha }}}+\sum_{\alpha \in I\;}\;\lambda_{P}^{\alpha }\cdot P_{*}^{\bar{\bar{\alpha }}}+\sum_{\alpha \in I\;}\;\lambda_{M}^{\alpha }M_{*}^{\bar{\bar{\alpha }}}\\ \;+\sum_{\alpha \in I\;}\;\lambda_{G}^{\alpha }G_{*}^{\bar{\bar{\alpha }}}+\sum_{\alpha \in I\;}\;\lambda_{T}^{\alpha }T_{*}^{\bar{\bar{\alpha }}}+\sum_{\alpha \in I\;}\;\lambda_{TG}^{\alpha }T_{G*}^{\bar{\bar{\alpha }}}=\sum_{\alpha \in I\;}\;\Lambda^{\bar{\bar{\alpha }}}\geq 0\;. \end{array}$$

Solution for the Lagrange multiplier coefficients to eliminate the material time derivatives provided

(50) $$\begin{array}{c} \sum_{\alpha \in I\;}\;S_{*}^{\bar{\bar{\alpha }}}-\sum_{\alpha \in I\;}\;\frac{1}{\theta^{\bar{\bar{\alpha }}}}E_{*}^{\bar{\bar{\alpha }}}+\sum_{\alpha \in I\;}\;\frac{1}{\theta^{\bar{\bar{\alpha }}}}v_{}^{\bar{\alpha }}\cdot P_{*}^{\bar{\bar{\alpha }}}\\ \;+\sum_{\alpha \in I\;}\;\frac{1}{\theta^{\bar{\bar{\alpha }}}}\left(\mu_{}^{\bar{\alpha }}+\psi_{}^{\bar{\alpha }}-\frac{v_{}^{\bar{\alpha }}\cdot v_{}^{\bar{\alpha }}}{2}+K_{E}^{\bar{\bar{\alpha }}}\right)M_{*}^{\bar{\bar{\alpha }}}\\ \;-\sum_{\alpha \in I\;}\;\frac{1}{\theta^{\bar{\bar{\alpha }}}}G_{*}^{\bar{\bar{\alpha }}}+\sum_{\alpha \in I\;}\;\frac{1}{\theta^{\bar{\bar{\alpha }}}}T_{*}^{\bar{\bar{\alpha }}}+\sum_{\alpha \in I\;}\;\frac{1}{\theta^{\bar{\bar{\alpha }}}}T_{G*}^{\bar{\bar{\alpha }}}=\sum_{\alpha \in I\;}\;\Lambda^{\bar{\bar{\alpha }}}\geq 0\;. \end{array}$$

With these coefficients, the time derivative of the deviation kinetic energy $K_{E}^{\bar{\bar{\alpha }}}$ survives in the inequality. For a porous media system, this term is very small and thus its presence does not impact the derivation. However, for a turbulent flow, this term is significant and should be addressed by making use of an $K_{*}^{\bar{\bar{\alpha }}}$ equation. We can include either Equation (24) or Equation (25) as an additional constraint in the entropy equation if it is included prior to identifying the Lagrange coefficients. However, we can alternatively use Equation (25) as an add-on to the entropy inequality as given in Equation (50) with the Lagrange coefficient $1/\theta^{\bar{\bar{\alpha }}}$ to obtain

(51) $$\begin{array}{c} \sum_{\alpha \in I\;}\;S_{*}^{\bar{\bar{\alpha }}}-\sum_{\alpha \in I\;}\;\frac{1}{\theta^{\bar{\bar{\alpha }}}}E_{*}^{\bar{\bar{\alpha }}}+\sum_{\alpha \in I\;}\;\frac{1}{\theta^{\bar{\bar{\alpha }}}}v_{}^{\bar{\alpha }}\cdot P_{*}^{\bar{\bar{\alpha }}}\\ \;+\sum_{\alpha \in I\;}\;\frac{1}{\theta^{\bar{\bar{\alpha }}}}\left(\mu_{}^{\bar{\alpha }}+\psi_{}^{\bar{\alpha }}-\frac{v_{}^{\bar{\alpha }}\cdot v_{}^{\bar{\alpha }}}{2}+K_{E}^{\bar{\bar{\alpha }}}\right)M_{*}^{\bar{\bar{\alpha }}}+\sum_{\alpha \in I\;}\;\frac{1}{\theta^{\bar{\bar{\alpha }}}}K_{*2}^{\bar{\bar{\alpha }}}\\ \;-\sum_{\alpha \in I\;}\;\frac{1}{\theta^{\bar{\bar{\alpha }}}}G_{*}^{\bar{\bar{\alpha }}}+\sum_{\alpha \in I\;}\;\frac{1}{\theta^{\bar{\bar{\alpha }}}}T_{*}^{\bar{\bar{\alpha }}}+\sum_{\alpha \in I\;}\;\frac{1}{\theta^{\bar{\bar{\alpha }}}}T_{G*}^{\bar{\bar{\alpha }}}=\sum_{\alpha \in I\;}\;\Lambda^{\bar{\bar{\alpha }}}\geq 0\;. \end{array}$$

After substitution of the definition of $K_{*2}^{\bar{\bar{\alpha }}}$ as given by the first equality in Equation (25) and rearrangement of terms, this becomes

(52) $$\begin{array}{c} \sum_{\alpha \in I\;}\;S_{*}^{\bar{\bar{\alpha }}}-\sum_{\alpha \in I\;}\;\frac{1}{\theta^{\bar{\bar{\alpha }}}}{\langle E_{\alpha *}-v_{\alpha }\cdot P_{\alpha *}+\frac{v_{\alpha }\cdot v_{\alpha }}{2}M_{\alpha *}\rangle }_{\Omega_{\alpha },\Omega_{}}\\ \;+\sum_{\alpha \in I\;}\;\frac{1}{\theta^{\bar{\bar{\alpha }}}}\left(\mu_{}^{\bar{\alpha }}+\psi_{}^{\bar{\alpha }}\right)M_{*}^{\bar{\bar{\alpha }}}\\ \;-\sum_{\alpha \in I\;}\;\frac{1}{\theta^{\bar{\bar{\alpha }}}}G_{*}^{\bar{\bar{\alpha }}}+\sum_{\alpha \in I\;}\;\frac{1}{\theta^{\bar{\bar{\alpha }}}}T_{*}^{\bar{\bar{\alpha }}}+\sum_{\alpha \in I\;}\;\frac{1}{\theta^{\bar{\bar{\alpha }}}}T_{G*}^{\bar{\bar{\alpha }}}=\sum_{\alpha \in I\;}\;\Lambda^{\bar{\bar{\alpha }}}\geq 0\;. \end{array}$$

This is noteworthy because we can identify $E_{\theta \alpha *}$ as the microscale internal energy equation given by

(53) $$E_{\theta \alpha *}:=E_{\alpha *}-v_{\alpha }\cdot P_{\alpha *}+\frac{v_{\alpha }\cdot v_{\alpha }}{2}M_{\alpha *}\;.$$

The CEI in general then takes the form

(54) $$\begin{array}{c} \sum_{\alpha \in I\;}\;S_{*}^{\bar{\bar{\alpha }}}-\sum_{\alpha \in I\;}\;\frac{1}{\theta^{\bar{\bar{\alpha }}}}E_{\theta *}^{\bar{\bar{\alpha }}}+\sum_{\alpha \in I\;}\;\frac{1}{\theta^{\bar{\bar{\alpha }}}}\left(\mu_{}^{\bar{\alpha }}+\psi_{}^{\bar{\alpha }}\right)M_{*}^{\bar{\bar{\alpha }}}\\ \;-\sum_{\alpha \in I\;}\;\frac{1}{\theta^{\bar{\bar{\alpha }}}}G_{*}^{\bar{\bar{\alpha }}}+\sum_{\alpha \in I\;}\;\frac{1}{\theta^{\bar{\bar{\alpha }}}}T_{*}^{\bar{\bar{\alpha }}}+\sum_{\alpha \in I\;}\;\frac{1}{\theta^{\bar{\bar{\alpha }}}}T_{G*}^{\bar{\bar{\alpha }}}=\sum_{\alpha \in I\;}\;\Lambda^{\bar{\bar{\alpha }}}\geq 0\;, \end{array}$$

where

(55) $$E_{\theta *}^{\bar{\bar{\alpha }}}={\langle E_{\alpha *}-v_{\alpha }\cdot P_{\alpha *}+\frac{v_{\alpha }\cdot v_{\alpha }}{2}M_{\alpha *}\rangle }_{\Omega_{\alpha },\Omega_{}}\;.$$

Note that, although Equation (53) applies at the small scale,

(56) $$E_{\theta *}^{\bar{\bar{\alpha }}}\neq E_{*}^{\bar{\bar{\alpha }}}-v_{}^{\bar{\alpha }}\cdot P_{*}^{\bar{\bar{\alpha }}}+\frac{v_{}^{\bar{\alpha }}\cdot v_{}^{\bar{\alpha }}}{2}\cdot M_{*}^{\bar{\bar{\alpha }}}\;.$$

The proper definition of the thermal energy equation at the macroscale has been a source of error in the study of diffusive processes (e.g., [29]) as has been discussed elsewhere [30].

These results provide the components needed to treat deviation kinetic energy as a leading-order quantity, which will be of use in extending TCAT to turbulent flow conditions. Examples of where this will be important are plentiful in the geosciences, including land–atmosphere interaction and sediment transport in streams, rivers, and oceans.

## 6. Leveraging Existing Entropy Production Equations

As previously noted, SEI expressions in flux–force form have been derived for several TCAT model hierarchies. It is stated that these legacy entropy production expressions can be used to produce closure relations that enable the formulation of solvable models. The purpose of this section is to provide a simple example to show how closure relations can be determined from an SEI.

Let us consider single-fluid-phase flow through porous media. The index set of entities is I={w,s,ws} denoting a water and solid phase, and water–solid interface, respectively. An REV is assumed, classical irreversible thermodynamics relied upon, and compositional effect are assumed to be unimportant. This system has been examined and a CEI and SEI have been produced [5,9]. We will focus this example on a particular form of the SEI that is available (Gray and Miller [5], Equation (9.63)). The example SEI can be written as

(57) $$\begin{array}{c} \frac{1}{\theta }\left(\epsilon^{\bar{\bar{w}}}t^{\bar{\bar{w}}}+\epsilon^{\bar{\bar{w}}}p_{}^{w}I\right):d^{\bar{\bar{w}}}+\frac{1}{\theta }\left(\epsilon^{\bar{\bar{s}}}t^{\bar{\bar{s}}}-\epsilon^{\bar{\bar{s}}}t^{s}\right):d^{\bar{\bar{s}}}\\ +\frac{1}{\theta }\left[\epsilon^{\bar{\bar{ws}}}t^{\bar{\bar{ws}}}-\epsilon^{\bar{\bar{ws}}}\gamma^{ws}\left(I-G^{ws}\right)\right]:d^{\bar{\bar{ws}}}\\ +\frac{1}{\theta }\left[\nabla \left(\epsilon^{\bar{\bar{w}}}p_{}^{w}\right)-\epsilon^{\bar{\bar{w}}}\rho^{w}\nabla \left(\mu_{}^{\bar{w}}+\psi_{}^{\bar{w}}\right)-\epsilon^{\bar{\bar{w}}}\rho^{w}g_{}^{\bar{w}}+\overset{w\to ws}{T}\right]\cdot \left(v_{}^{\bar{w}}-v_{}^{\bar{s}}\right)\\ -\frac{1}{\theta }\left\{\nabla \cdot \left[\left(I-G^{ws}\right)\epsilon^{\bar{\bar{ws}}}\gamma^{ws}\right]+\overset{w\to ws}{T}+\overset{s\to ws}{T}\right\}\cdot \left(v_{}^{\bar{ws}}-v_{}^{\bar{s}}\right)\\ -\frac{1}{\theta }\left[p_{w}^{ws\;}+{\langle n_{s}\;\cdot \;t_{s}\cdot n_{s}\rangle }_{\Omega_{ws},\Omega_{ws}}+\gamma^{ws}J_{s}^{\;ws}\right]\frac{D^{\bar{s}}\epsilon^{\bar{\bar{s}}}}{Dt}=\sum_{\alpha \in I\;}\;\Lambda^{\bar{\bar{\alpha }}}\geq 0\;, \end{array}$$

where $p_{}^{w}$ is pressure of the water phase, $G^{ws}$ is the orientation tensor of the interface, $\mu_{}^{\bar{w}}$ is the chemical potential of the *w* phase, $\psi_{}^{\bar{w}}$ is the gravitational potential of the *w* phase, $p_{w}^{ws\;}$ is the pressure of the *w* phase averaged over the interface, $n_{s}$ is the unit vector outward normal to the solid phase, $\gamma^{ws}$ is the interfacial tension of the interface, and $J_{s}^{\;ws}$ is twice the mean curvature of the interface.

The general SEI has been simplified in writing Equation (57) for the case of isothermal conditions, no mass exchange, slow flow, and a massless interface, which comprise a set of secondary restrictions that were applied after a general SEI was derived and are not in general necessary. This is an example of how a general CEI, obtained by substituting the appropriate macroscale equations into Equation (50), can yield a set of different SEIs. As a further example of this notion, we could assume a rigid, incompressible solid phase, and choose to ignore the effects of the interface. These additional secondary restrictions would further simplify the resultant SEI to a form given by

(58) $$\begin{array}{c} \frac{1}{\theta }\left(\epsilon^{\bar{\bar{w}}}t^{\bar{\bar{w}}}+\epsilon^{\bar{\bar{w}}}p_{}^{w}I\right):d^{\bar{\bar{w}}}\\ +\frac{1}{\theta }\left[\nabla \left(\epsilon^{\bar{\bar{w}}}p_{}^{w}\right)-\epsilon^{\bar{\bar{w}}}\rho^{w}\nabla \left(\mu_{}^{\bar{w}}+\psi_{}^{\bar{w}}\right)-\epsilon^{\bar{\bar{w}}}\rho^{w}g_{}^{\bar{w}}+\overset{w\to ws}{T}\right]\cdot \left(v_{}^{\bar{w}}-v_{}^{\bar{s}}\right)\\ =\sum_{\alpha \in I\;}\;\Lambda^{\bar{\bar{\alpha }}}\geq 0\;, \end{array}$$

Equation (58) contains the sum to two flux–force products. The forces are $d^{\bar{\bar{w}}}$ and $v_{}^{\bar{w}}-v_{}^{\bar{s}}$, and the fluxes are terms that multiply these quantities. Both of the forces and both of the fluxes vanish at equilibrium. It is known that away from equilibrium, these flux–force pairs may produce entropy. Each of these pairs must independently satisfy this entropy production condition, because the fluxes are a set with independent members and so too are the forces.

TCAT uses the entropy production requirement in SEIs, such as Equation (58), as constraints, or permissibility conditions, on the development of closure relations needed to formulate closed, solvable models. Let us consider the first line in this SEI. We know that this line cannot be less than zero, so the approximation for the stress tensor must ensure this is the case. For porous medium systems at the macroscale, momentum transfer between the fluid phase and the solid phase at slow flow tends to overwhelm viscous stress effects. Because of this, a zero-order closure provides a good approximation for macroscale porous medium flow, which is equivalent to treating the fluid phase as macroscopically inviscid. This macroscopic condition means that the average velocity of the system is not affected by the boundary of the domain—an advective front in a one-dimensional flow field with a homogeneous domain will be invariant in the directions normal to the direction of flow. With this approximation, the stress tensor becomes

(59) $$t^{\bar{\bar{w}}}=-p_{}^{w}I\;.$$

A consequence of Equation (59) is that the first line in Equation (58) is always zero, which satisfies the entropy inequality for any $d^{\bar{\bar{w}}}$.

The second line in Equation (58) must also be satisfied. $\overset{w\to ws}{T}$ is an important term in this line, and it is unknown from any conservation, thermodynamic, or potential equation in the formulation. In a conjugate flux–force approximation, it is posited that the flux depends solely on the force appearing in this line. Any permissible approximation for the flux must be of a form such that the second line in Equation (58) is non-negative under all conditions. A conjugate first-order closure approximation for the flux is

(60) $$\nabla \left(\epsilon^{\bar{\bar{w}}}p_{}^{w}\right)-\epsilon^{\bar{\bar{w}}}\rho^{w}\nabla \left(\mu_{}^{\bar{w}}+\psi_{}^{\bar{w}}\right)-\epsilon^{\bar{\bar{w}}}\rho^{w}g_{}^{\bar{w}}+\overset{w\to ws}{T}={\hat{R}}^{w}\cdot \left(v_{}^{\bar{w}}-v_{}^{\bar{s}}\right)\;,$$

where ${\hat{R}}^{w}$ is a second-rank, symmetric, positive semi-definite resistance tensor. Substituting Equations (59) and (60) into Equation (58) yields

(61) $$\left(v_{}^{\bar{w}}-v_{}^{\bar{s}}\right)\cdot {\hat{R}}^{w}\cdot \left(v_{}^{\bar{w}}-v_{}^{\bar{s}}\right)=\sum_{\alpha \in I\;}\;\Lambda^{\bar{\bar{\alpha }}}\geq 0\;,$$

which must hold according to the properties assigned to ${\hat{R}}^{w}$ and provides an expression for the entropy density production rate of the system subject to derivative restrictions and approximations. Equation (60) can in turn be used to formulate a closed solvable model for single-fluid-phase flow.

This example illustrates how available SEIs can be used to produce closure relations and in turn closed solvable models. Since a significant, and growing, set of such expressions are available, existing TCAT results can be leveraged simply to develop new models for a wide range of systems. Such results can also be used to evaluate *ad hoc* models developed without the rigor of TCAT to evaluate if the forms in common use meet the entropy production constraint needed to assure validity. The deviation kinetic energy results derived above would enable the inclusion of deviation kinetic energy in an entropy production equation without the neglect of terms such as the material derivative of this quantity, which has been commonly done for porous medium applications [5]. This result could be used to evaluate if commonly used closure relations for applications such as sediment transport are consistent with the second law entropy production rate expression. Previously published results have concluded that some multiphase models in common use violate the second law of thermodynamics [31], although important issues of even a precise definition and appropriate scale at which turbulence should be modeled remain open issues (e.g., [32]). Furthermore, entropy production expressions provide a means to resolve the rate of entropy production as a function of space and time in a rigorous fashion, which can in turn be used to add an appropriate level of dissipation to numerical approximations, such as the finite element method (e.g., [33,34]).

## 7. Conclusions

We conclude the following:

1. TCAT is a mature framework for developing scale-consistent models of transport phenomena for multiphase system based upon the rate of entropy production.
2. While TCAT has many desirable attributes, the underlying mathematical methods relied upon are several in number and can be complicated for those not familiar with the approaches.
3. The key results from TCAT are entropy inequality expressions, which can span multiple pages of heavily adorned symbols that can inhibit understanding and use.
4. Some mathematical machinery relied upon in TCAT is summarized along with how such methods contribute to the theory.
5. The overall process to model building is described at a component level to provide a basic understanding of the approach.
6. Available component results and model hierarchies are summarized.
7. An extension of TCAT methods to turbulent systems is considered and useful deviation kinetic energy components for such an extension are formulated, and the consequences of using these components for entropy production is shown.
8. An example is provided to show how entropy production can be used to provide closure relation approximations, thereby leveraging the growing set of TCAT model hierarchies already derived.

## Figures and Tables

**Table 1:** Mathematical foundational elements of TCAT.

Element	Use	References
Curvilinear coordinates	Microscale description of entity domains and boundaries	[5,6]
Averaging operators	General change of scale operators for entities of varying dimensions and weighting	[4,5]
Generalized functions	Formulation of divergence, gradient, and transport theorems to relate averages of derivatives to derivatives of averages for all entities	[5,6]
Variational methods	Derivation of thermodynamic equilibrium conditions for multiphase systems	[5,17,18,19]
Differential geometry	Geometric characteristics of entities and boundaries	[5,6]

**Table 2:** TCAT model hierarchies of existing CEI and SEI expressions.

Entities	Composition	Scale	Reference
One fluid, one solid, and one interface	Entity based	Macroscale in three dimensions	[5,9]
One fluid, one solid, and one interface	Entity based	Megascale in three dimensions	[10]
One fluid, one solid, and one interface	Species based for mass and entity based for momentum and energy	Macroscale in three dimensions	[5,9]
One fluid, one solid, and one interface	Species based for mass and momentum and entity based for energy	Macroscale in three dimensions	[9]
Two fluids, one solid, three interfaces, and one common curve	Entity based	Macroscale in three dimensions	[5,11]
Two fluids, one solid, three interfaces, and one common curve	Species based for mass and entity based for momentum and energy	Macroscale in three dimensions	[13]
Two fluids, one solid, three interfaces, and one common curve	Species based for mass and entity based for momentum and energy	Macroscale in two dimensions and megascale in one dimension	[12]

## Notation

### Roman Letters

d	rate of strain tensor, $d=\frac{1}{2}\left[\nabla v+{\left(\nabla v\right)}^{{}^{T}}\right]$
*E*	internal energy density
E	conservation of energy equation
$E_{\theta }$	conservation of internal energy equation
*F*	quantity to be minimized
*f*	arbitrary continuous function
$f_{n}$	a functional
G	body force potential balance equation
G	orientation tensor
g	gravitational acceleration vector
I	unit tensor
$I^{\prime }$	unit tensor in a surface
$I^{\prime \prime }$	unit tensor in a common curve
I	index set of entities
$I_{C}$	index set of common curve entities
$I_{c\alpha }^{-}$	index set of entities connected to entity α that are of one dimension lower than entity α
$I_{I}$	index set of interface entities
$I_{P}$	index set of phase entities
$J_{s}^{ws}$	twice the mean curvature of the interface between the solid and wetting phases
K	deviation kinetic energy equation
$K_{E}$	deviation kinetic energy per unit mass due to velocity fluctuations
$\underset{\kappa \to \alpha }{M}$	microscale mass transfer rate from the κ entity to the α entity
$\overset{\kappa \to \alpha }{M}$	macroscale mass transfer rate from the κ entity to the α entity
M	conservation of mass equation
$n_{\alpha }$	unit normal vector on the boundary of entity α oriented to be positive outward
P	conservation of momentum equation
*p*	pressure
IR	the set of real numbers
${\hat{R}}^{w}$	resistance tensor
r	position vector
r	general integration variable
S	entropy balance equation
*s*	solid phase entity index
$\underset{\kappa \to \alpha }{T}$	microscale transfer of momentum from entity κ to entity α due to stress
$\overset{\kappa \to \alpha }{T}$	macroscale transfer of momentum from entity κ to entity α due to stress and deviation from mean processes
T	thermodynamic equation
$T_{G}$	material derivative of the body force potential equation
*t*	time
t	stress tensor
u	vector of unknown functions
v	velocity
*W*	weighting function used in averaging
x	position vector

### Greek Letters

α	member of I referring to phase, interface, or common curve α
γ	interfacial tension
$\epsilon^{\bar{\bar{\alpha }}}$	specific entity measure
θ	temperature
$\theta^{\bar{\bar{\alpha }}}$	entropy-weighted macroscale temperature of entity α
κ	member of I referring to phase, interface, or common curve κ
Λ	entropy production rate
μ	chemical potential
ξ	microscale position vector
ρ	mass density
ψ	body force potential
$\Omega_{}$	spatial domain of the averaging volume
$\Omega_{\alpha }$	domain occupied by entity α within the averaging volume

### Subscripts (for Microscale) and Superscripts (for Macroscale)

*s*	solid-phase qualifier, scalar qualifier
*w*	wetting-phase qualifier
ws	interface qualifier for surface between the wetting phase and solid phase
α	entity qualifier
*	qualifier for particular form of equation, which can be further qualified with an integer

### Symbols

$\tilde{\;\;}$	above a variable denotes the deviation between the microscale quantity and the macroscale average quantity
${}^{\bar{\;\;}}$	above a superscript refers to a density-weighted macroscale average
${}^{\bar{\bar{\;\;}}}$	above a superscript refers to a uniquely defined macroscale average
${\langle f_{\alpha }\rangle }_{\Omega_{b},\Omega_{c},W}$	$=\left(\int_{\Omega_{b}}^{}Wf_{\alpha }\;dr\right)/\left(\int_{\Omega_{c}}^{}W\;dr\right)$, general average of a microscale property $f_{\alpha }$
${\langle f_{\alpha }\rangle }_{\Omega_{b},\Omega_{c}}$	$={\langle f_{\alpha }\rangle }_{\Omega_{b},\Omega_{c},1}$
$f_{\alpha }^{\beta }$	$={\langle f_{\alpha }\rangle }_{\Omega_{\beta },\Omega_{\beta }}$, general average
$f^{\alpha }$	$={\langle f_{\alpha }\rangle }_{\Omega_{\alpha },\Omega_{\alpha }}$, intrinsic average
$f_{\alpha }^{\bar{\beta }}$	$={\langle f_{\alpha }\rangle }_{\Omega_{\beta },\Omega_{\beta },\rho_{\alpha }}$, general density-weighted average
$f^{\bar{\alpha }}$	$={\langle f_{\alpha }\rangle }_{\Omega_{\alpha },\Omega_{\alpha },\rho_{\alpha }}$, intrinsic density-weighted average
$D^{\bar{\alpha }}/Dt$	macroscale material derivative with macroscale velocity $v_{}^{\bar{\alpha }}$, $D^{\bar{\alpha }}/Dt=\partial /\partial t+v_{}^{\bar{\alpha }}\cdot \nabla$
∇	spatial gradient operator
∇·	spatial divergence operator
$\nabla^{\prime }$	microscale surficial gradient operator on a microscale interface
$\nabla^{\prime }\cdot$	microscale surface divergence vector
$\nabla^{\prime \prime }$	microscale gradient operator along a curve
$\nabla^{\prime \prime }\cdot$	microscale divergence operator for a curve
∂/∂t	time derivative with all spatial coordinates fixed
$\partial^{\prime }/\partial t$	partial time derivative fixed to point on a surface of a function dependent on microscale spatial coordinates and time
$\partial^{\prime \prime }/\partial t$	partial time derivative fixed to a point on a curve of a function dependent on microscale spatial coordinates and time
${}^{T}$	transpose operator

## Abbreviations

CEI	constrained entropy inequality
EI	entropy inequality
REV	representative elementary volume for averaging from the microscale to the macroscale such that average values are stable and independent of the averaging scale
SEI	simplified entropy inequality
TCAT	thermodynamically constrained averaging theory
